# Insensitive ionic bio-energetic materials derived from amino acids

**DOI:** 10.1038/s41598-017-12812-7

**Published:** 2017-10-06

**Authors:** Lei Zhang, Kang-Xiang Song, Zhang Zhang, Wen-Li Yuan, Nanrong Zhao, Song Qin, Ling He, Guo-Hong Tao

**Affiliations:** College of Chemistry, Sichuan University, Chengdu, 610064 China

## Abstract

Energetic salts/ionic liquids have received increasing attention as fascinating energetic materials, and the use of renewable compounds is a promising approach to developing energetic materials. Until recently, biomolecules have been used as raw materials to develop neutral energetic compounds, whereas research focused on ionic energetic materials obtained from natural bio-renewable frameworks is scarce. This work systematically investigates ionic bio-energetic materials (IBEMs) derived from sustainable natural amino acids. In addition to combustibility, high density, good thermal stability, and one-step preparation, these IBEMs demonstrated apparent hypotoxicity and insensitivity. Moreover, a theoretical examination was performed to explore their appropriate properties. The intriguing results of this study indicates that IBEMs are potential bio-based energetic materials.

## Introduction

**Figure 1:**
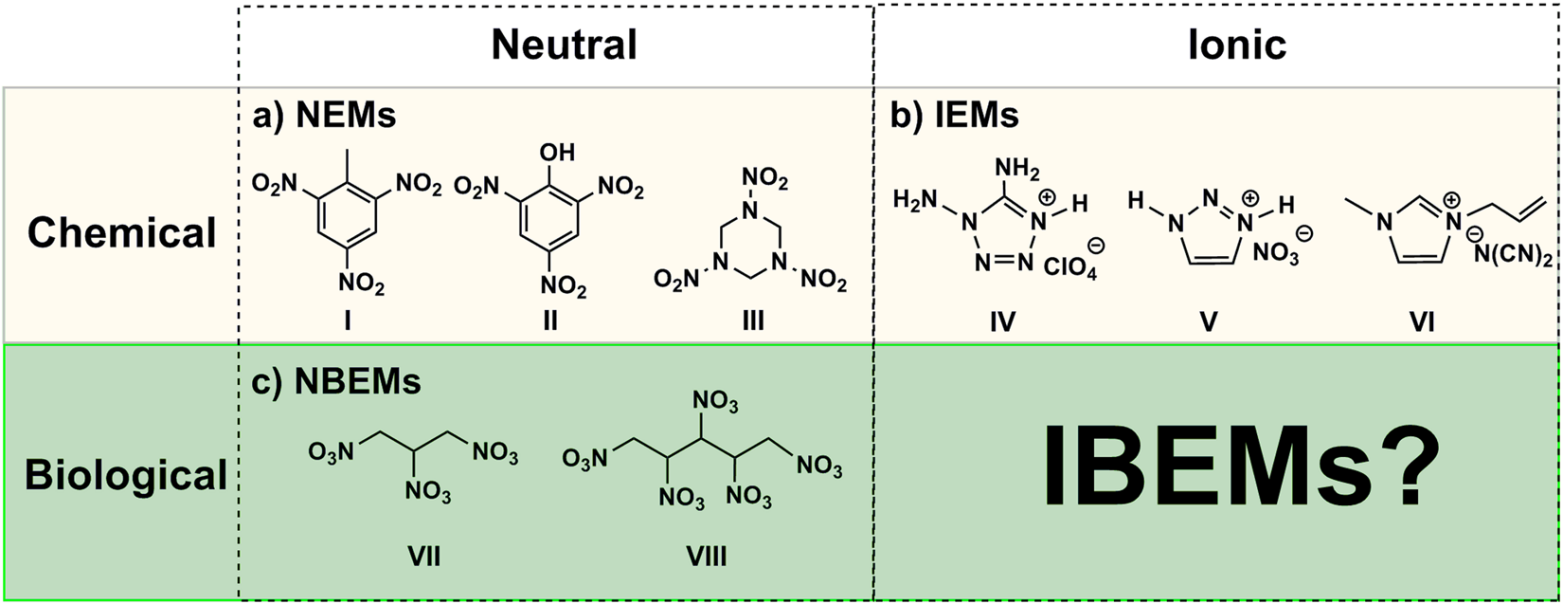
(**a**) Structures of NEMs from chemicals. I: TNT, II: PA, III RDX. (**b**) Structures of common IEMs from chemicals. IV: 1,5-Diamino-4-*H*−1,2,3,4-tetrazolium perchlorate, V: 1,2,3-triazolium nitrate, VI: 1-Allyl-3-methylimidazolium dicyanamide. (**c**) Structures of common NBEMs from biomolecules. VII: NG, VIII: XPN.

Energetic materials with large amounts of stored energy that can be released under specific conditions are of crucial importance and have many uses, including as explosives, propellants and pyrotechnics^1–7^. The most widely applied energetic materials are neutral compounds, such as 2,4,6-trinitrotoluene (TNT), 2,4,6-trinitrophenol (TNP), 1,3,5-trinitro-1,3,5-triazacyclohexane (RDX) and octahydro-1,3,5,7-tetranitro-1,3,5,7-tetrazocine (HMX)^8^, (Fig. 1), although these compounds suffer from their requirement for a multistep synthesis and their sensitivity^9,10^. In addition, the starting materials, e.g., methylbenzene, are non-renewable petrochemicals. Development and use of renewable starting materials has significant importance in sustainable development and recovery of waste resources^11,12^, and use of bio-renewable feed can avoid the complicated coal/petro-chemical process of precursors^13–15^. Biomolecules have been used as raw materials for the preparation of neutral bio-energetic materials (NBEMs), which are composed mainly of sugar alcohol-based energetic compounds (including nitroglycerin (NG), xylitol pentanitrate (XPN), mannitol hexanitrate (MHN), and nitrocellulose (NC))^16^ (Fig. 1). Bio-energetic materials have received renewed attention in recent years^17–21^. Certain NBEMs derived from sugar alcohols and glycine were reported by Klapötke *et al*.^22,23^. However, studies on this bio-based species focus primarily on neutral compounds.

Ionic energetic materials (IEMs) including energetic salts and energetic ionic liquids have drawn extensive attention due to their designability and little/no vapor toxicity compared with the traditional NEMs^24–27^. IEMs, which are usually composed of heterocyclic cations (e.g., imidazolium, triazolium, tetrazolium cation, etc.) and energetic anions (e.g., nitrate (NO_3_^−^), perchlorate (ClO_4_^−^), dicyanamide (DCA^−^) anion and bulky anions with one or more energetic groups, such as −NH_2_, −N_3_, and −CN, etc.)^28–32^, have demonstrated their excellent performance and great development potential in propellants and explosives (Fig. 1), but the formation materials are too sensitive and contain too many functional groups, which make their synthesis highly difficult and expensive^33^. Many IEM tasks remain to facilitate the development of promising energetic materials involving careful selection of precursors and synthetic protocols.

Amino acids, which are the building blocks of peptides and proteins, are common and low-cost bio-renewable molecules with amino (‒NH_2_) and carboxylic (‒COOH) functional groups. Production of amino acids can be accomplished by hydrolysis of proteins, of which approximately 10^8^ tons are wasted each year^34^. Amino acids belong to the natural carbon pool, which is the intrinsic source of fuels and propellants. For example, glycine was previously incorporated as a fuel in the HAN-based propellant (HAN: hydroxylammonium nitrate) and also used as a raw material to construct neutral highly energetic oxidizers^19,22,35–37^. The energetic properties of amino acid salts/ionic liquids directly derived from amino acids have been rarely examined, although their characteristics as catalysts, absorbents and chiral reagents have been studied^38–43^. The possibility of developing ionic bio-energetic materials (IBEMs) from amino acid frameworks is systematically discussed in this work.

## Results and Discussion

**Figure 2:**
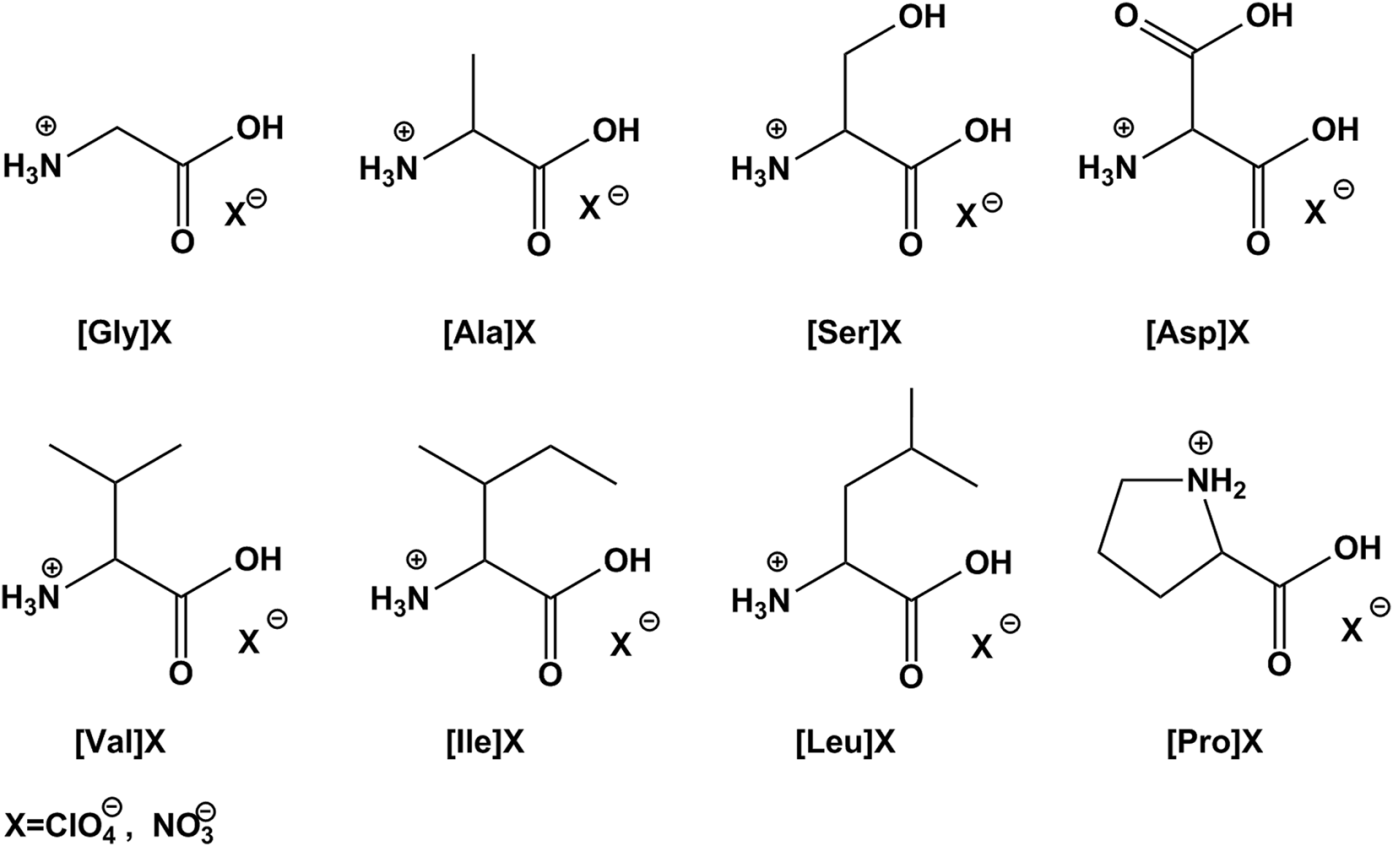
Structures of energetic salts/ionic liquids [AA]ClO_4_ and [AA]NO_3_ derived from amino acids.

A series of IBEMs, including amino acid salts/ionic liquids [AA]ClO_4_ and [AA]NO_3_ (Fig. 2), were synthesized from natural amino acids and oxygen-rich perchloric acid (HClO_4_) or nitric acid (HNO_3_) in one step without any byproducts^38^. The synthesis protocol is a typical “atom economic reaction” in water at ambient temperature and pressure. This simple procedure and the use of biomaterials have the advantages of energy conservation, an abundant renewable source and low costs^34^. The resulting IBEMs [AA]ClO_4_ and [AA]NO_3_ could serve as a novel framework for safer bio-energetic materials. These materials were identified by nuclear magnetic resonance spectrometry (NMR) and Fourier transform infrared spectrometry (FTIR). Colorless crystals of [Gly]ClO_4_ and [Ala]NO_3_ suitable for single-crystal X-ray analysis were also obtained.

**Figure 3:**
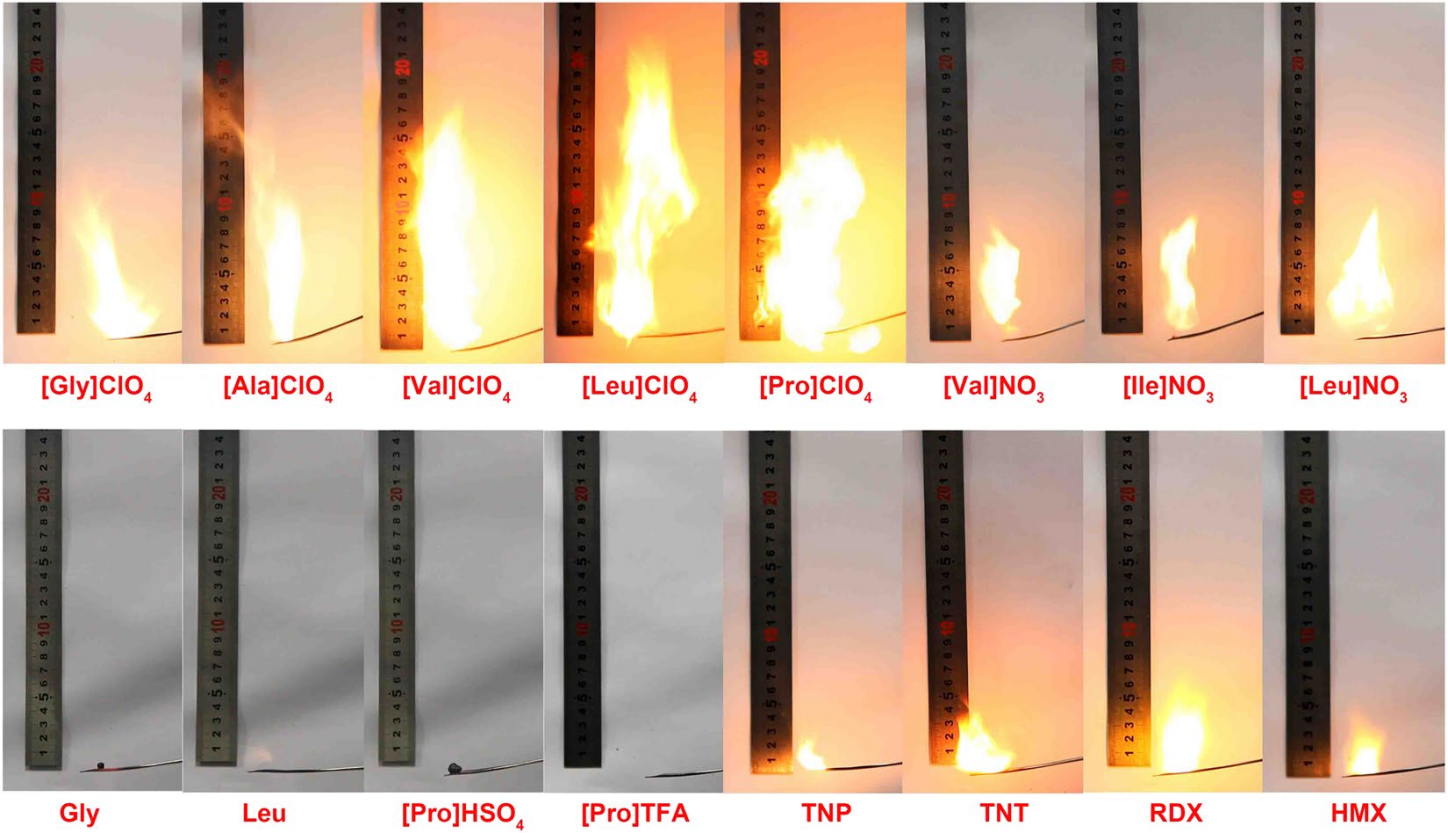
Combustion tests of 10 mg samples.

The combustibility test is an essential and simple test used to obtain a first impression of the energetic behavior of a compound^44^. The combustible characteristics of IBEMs, amino acids and several conventional NEMs were examined by heating 10 mg samples with a flame in air. The two IBEMs [AA]ClO_4_ and [AA]NO_3_ in this study can be ignited and display good combustibility (Fig. 3). Stable combustion was sustained until all samples were exhausted. It can be observed from the set of combustion images (Figures S6–S10) that [AA]ClO_4_ exhibited quick and violent burning with notably intense fire. This fierce combustion might result from the high reactivity of ClO_4_^−^ and the good oxygen balance of [AA]ClO_4_. Sustained and mild combustion was observed when [AA]NO_3_ was burned (Figures S11–S13). In contrast, eight amino acids used as precursors in this work were difficult to ignite or produced faint combustion with tiny flames. The improved combustibility characteristic of [AA]ClO_4_ and [AA]NO_3_ should originate from the energetic anion species. Thus, the oxygen-rich energetic anions ClO_4_^−^ and NO_3_^−^ are favorable candidates for constructing IBEMs. Additionally, not all anions can improve the combustion of amino acid salts/ionic liquids, similar to the results from the contrast experiments. For example, prolinium hydrochloride ([Pro]Cl), prolinium hydrobromide ([Pro]Br), prolinium trifluoroacetate ([Pro]TFA), prolinium bisulfate.

([Pro]HSO_4_) and prolinium trifluoromethanesulfonate ([Pro]OTf) were all assessed under the same condition, and all present results analogous to those of the amino acids, which indicates that the corresponding Cl^−^, Br^−^, TFA^−^, HSO_4_^−^ and OTf^−^ anions are not suitable for designing IBEMs. The combustibility performances of TNT, TNP, RDX and HMX were also tested and showed sustainable combustion with a mild flame, corresponding to their popular applications in propellants and explosives. As a comparison, the combustion of most IBEMs in this work is clearly more vigorous than that of TNT, TNP, RDX and HMX.

**Figure 4:**
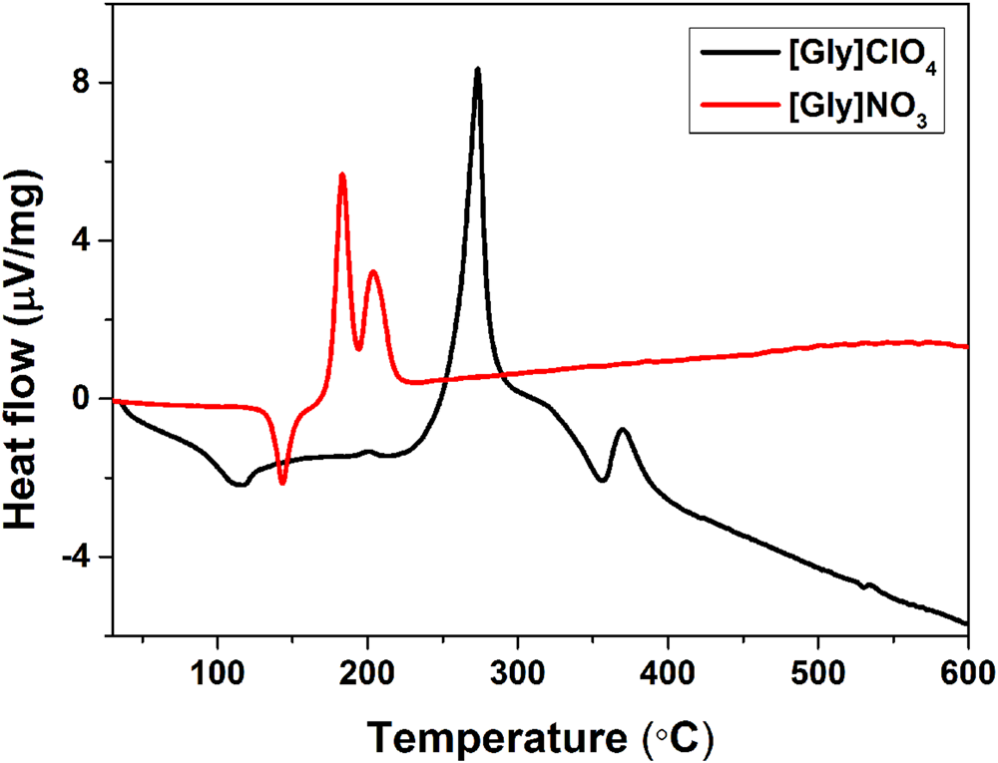
DTA profile displaying the heat flow of [Gly]ClO_4_ and [Gly]NO_3_.

Thermal stability is vital to the performance of energetic materials. [AA]ClO_4_ and [AA]NO_3_ are thermally stable at room temperature and can be maintained without any decomposition for more than two years. [AA]ClO_4_ exhibit good thermal stability with decomposition temperatures (*T*_d_) greater than 210 °C, which exceed the criterion of 200 °C^45,46^ (Table 1). The highest thermal stability out of all samples is found for [Gly]ClO_4_ (263 °C). The *T*_d_ values of [AA]NO_3_ are in the range of 125–183 °C, which are far beyond that of the typical NBEM and NG (50 °C). The melting points (*T*_m_) or glass transition temperatures (*T*_g_) of [AA]ClO_4_ and [AA]NO_3_ are between −46 °C and 159 °C. The initial melting point analysis reveals that [AA]ClO_4_ materials generally have significantly lower melting points than their nitrate analogues, among which many can be classified as ionic liquids. The thermal data also showed that certain IBEMs display a liquidus range at temperatures above 150 °C. The low *T*_m_ and good thermal stability indicate that these materials are easily molded, similar to TNT. Differential thermal analysis (DTA) is a routine method used to characterize energetic materials^17,18^. Heat flow was measured under a nitrogen environment at a heating rate of 10 °C min^−1^. The DTA profile of [Gly]ClO_4_ and [Gly]NO_3_ is illustrated in Fig. 4. A notably large and sharp exothermic peak of [Gly]ClO_4_ is observed at a peak temperature of approximately 273 °C, which could be related to the oxidation-reduction reaction between glycine cation and ClO_4_^−^. The DTA curve of [Gly]NO_3_ shows a sharp exothermic peak near 182 °C and a sharp endothermic peak near 145 °C due to [Gly]NO_3_ melting, which corresponds to the melting point (149 °C) observed by DSC. [Gly]ClO_4_ displays a higher exothermic temperature than [Gly]NO_3_, probably because ClO_4_^−^ is more stable than NO_3_^−^. The DTA curves of the remaining [AA]X are presented in the Supplementary Information.

**Table 1:** Physicochemical properties of IBEMs and reference compounds.

Compound	*ρ* ^a^	OB_CO_^b^	*T*_m_/*T*_g_^c^	*T*_d_^d^	−Δ_f_ *H*°^e^	−Δ_c_ *H*°^f^	*P* ^g^	*D* ^h^	*IS* ^i^	*FS* ^j^
[Gly]ClO_4_	1.89	13.67	103	263	4.22	5.29/5.49	32.26	8470	>60	>360
[Ala]ClO_4_	1.69	−4.22	88	246	4.22	8.17/8.48	17.88	6520	>60	>360
[Ser]ClO_4_	1.66	3.89	/−46	221	4.39	7.04	17.49	6486	>60	>360
[Asp]ClO_4_	1.68	3.43	/−26	245	4.82	6.92	16.34	6586	>60	>360
[Val]ClO_4_	1.47	−33.09	71	217	3.65	13.39/12.90	18.78	6994	>60	>360
[Ile]ClO_4_	1.46	−44.90	76	261	3.48	15.46/15.16	23.88	7903	>60	>360
[Leu]ClO_4_	1.46	−44.90	/	259	3.46	15.48/15.13	23.92	7911	>60	>360
[Pro]ClO_4_	1.57	−25.98	/	245	3.38	12.49/11.99	15.66	6246	>60	>360
[Gly]NO_3_	1.63	0	149	167	5.75	6.16	14.04	5844	>60	>360
[Ala]NO_3_	1.47	−21.04	158	178	5.59	9.69	15.48	6348	>60	>360
[Ser]NO_3_	1.55	−9.52	/	125	5.70	8.12	15.19	6179	>60	>360
[Asp]NO_3_	1.60	−8.16	98	183	6.02	7.83	22.9	7508	>60	>360
[Val]NO_3_	1.36	−53.29	134	169	4.70	15.74	15.43	6514	>60	>360
[Ile]NO_3_	1.32	−65.91	100	167	4.39	18.07	17.73	7058	>60	>360
[Leu]NO_3_	1.30	−65.91	159	192	4.37	18.09	17.31	7013	>60	>360
[Pro]NO_3_	1.48	−44.91	/	138	4.40	14.66	15.39	6316	>60	>360
NG^k^	1.59	24.66	14	50	1.67	6.73	/	7600	0.2	
TNT^k^	1.65	−24.66	80	295	0.12	15.00	19.53	6881	15	
TNP^k^	1.76	−3.49	122	300	0.98	12.47	26.5	7350	7.4	
NH_4_ClO_4_^k^	1.95	34.04		350	2.41				20	
NH_4_NO_3_^k^	1.73	20.00		170	4.43			5270	50	

^a^Density, 25 °C, g cm^−3^. ^b^Oxygen balance (based on CO), %. ^c^Melting point or glass-transition temperature, °C. ^d^Decomposition temperature, °C. ^e^Calculated negative enthalpy of formation, kJ kg^−1^. ^f^Calculated negative enthalpy of combustion, kJ g^−1^/Experimental negative enthalpy of combustion kJ g^−1^. ^g^Detonation pressure, GPa. ^h^Detonation velocity, m s^−1^. ^i^Impact sensitivity, J. ^j^Friction sensitivity, N. ^k^See ref.^8^.

A high density value is a profitable reference for energetic materials. Interestingly, the obtained perchlorates or nitrates are denser than their precursors. The densities of [Ala]ClO_4_ and [Ala]NO_3_ are 1.69 and 1.47 g cm^−3^, respectively, which are higher than that of alanine (1.42 g cm^−3^) (Table 1). The densities of [Ala]ClO_4_ (1.69 g cm^−3^), [Ser]ClO_4_ (1.66 g cm^−3^) and [Asp]ClO_4_ (1.68 g cm^−3^) are superior to those of TNT (1.65 g cm^−3^) and NG (1.59 g cm^−3^). The density of [Gly]ClO_4_ can reach 1.89 g cm^−3^, which outperforms the currently used energetic materials, such as RDX (1.82 g cm^−3^) and TNP (1.76 g cm^−3^), and is similar to HMX (1.91 g cm^−3^).

The standard enthalpy of formation (Δ_f_ *H*°) and heats of combustion (Δ_c_ *H*°) of [AA]ClO_4_ and [AA]NO_3_ were calculated using the Gaussian 09 suite of programs^47^, as described in our previous studies^48,49^. The theoretical data −Δ_f_ *H*° range is 3.46 to 6.02 kJ g^−1^, whereas that of [Leu]ClO_4_ is the lowest (3.46 kJ g^−1^). The theoretical data −Δ_c_ *H*° range is 5.29 to 18.09 kJ g^−1^. The experimental heats of combustion of six amino acid salts/ionic liquids ([Gly]ClO_4_, [Ala]ClO_4_, [Val]ClO_4_, [Ile]ClO_4_, [Leu]ClO_4_, and [Pro]ClO_4_) were determined to verify the accuracy of the theoretical results, and the relative error is less than 5%. Therefore, the theoretical results are in good agreement with the experimental results. Most of the −Δ_c_ *H*° values of IBEMs exceed that of NG (6.73 kJ g^−1^). The −Δ_c_ *H* ^0^ values of TNP (12.47 kJ g^−1^) are lower than those of [Val]ClO_4_, [Pro]ClO_4_, [Val]NO_3_ and [Pro]NO_3_. [Ile]ClO_4_ (15.46 kJ g^−1^), [Leu]ClO_4_ (15.48 kJ g^−1^), [Ile]NO_3_ (18.07 kJ g^−1^), and [Leu]NO_3_ (18.09 kJ g^−1^) possesses high −Δ_c_ *H*° values above that of TNT (15.00 kJ g^−1^). The calculated detonation pressures (*P*) of [AA]ClO_4_ and [AA]NO_3_ lie between 14.04 GPa and 32.26 GPa. The calculated detonation velocities (*D*) lie in the range from 5844 to 8470 m s^−1^, certain of which are greater than those of TNT (6881 m s^−1^), TNP (7350 m s^−1^) and NG (7600 m s^−1^). [Gly]ClO_4_ has the highest detonation pressure of 32.26 GPa and the highest detonation velocity of 8470 m s^−1^, which are comparable to those of RDX (34.9 GPa, 8748 m s^−1^). Because of the different structures of the amino acid cations, diverse oxygen balances (OB) are based on CO in a range of −65.91 to 13.67. Perchlorate displays good oxygen balance, and the OB_CO_ values of [Gly]ClO_4_ (13.67), [Ser]ClO_4_ (3.89) and [Asp]ClO_4_ (3.43) are positive. The sensitivities to impact of [AA]ClO_4_ and [AA]NO_3_ exceed 60 J. The two [AA]ClO_4_ and [AA]NO_3_ are stable at 360 N in the friction sensitivity test. The sensitivities of all IBEMs including [AA]ClO_4_ and [AA]NO_3_ are better than those of NBEM NG (0.2 J, 1 N). Based on the UN standard, all salts show both impact and friction insensitivity characteristics. The impact and friction sensitivities are remarkably good, especially for perchlorate salts because they are usually sensitive^29,30^.

**Figure 5:**
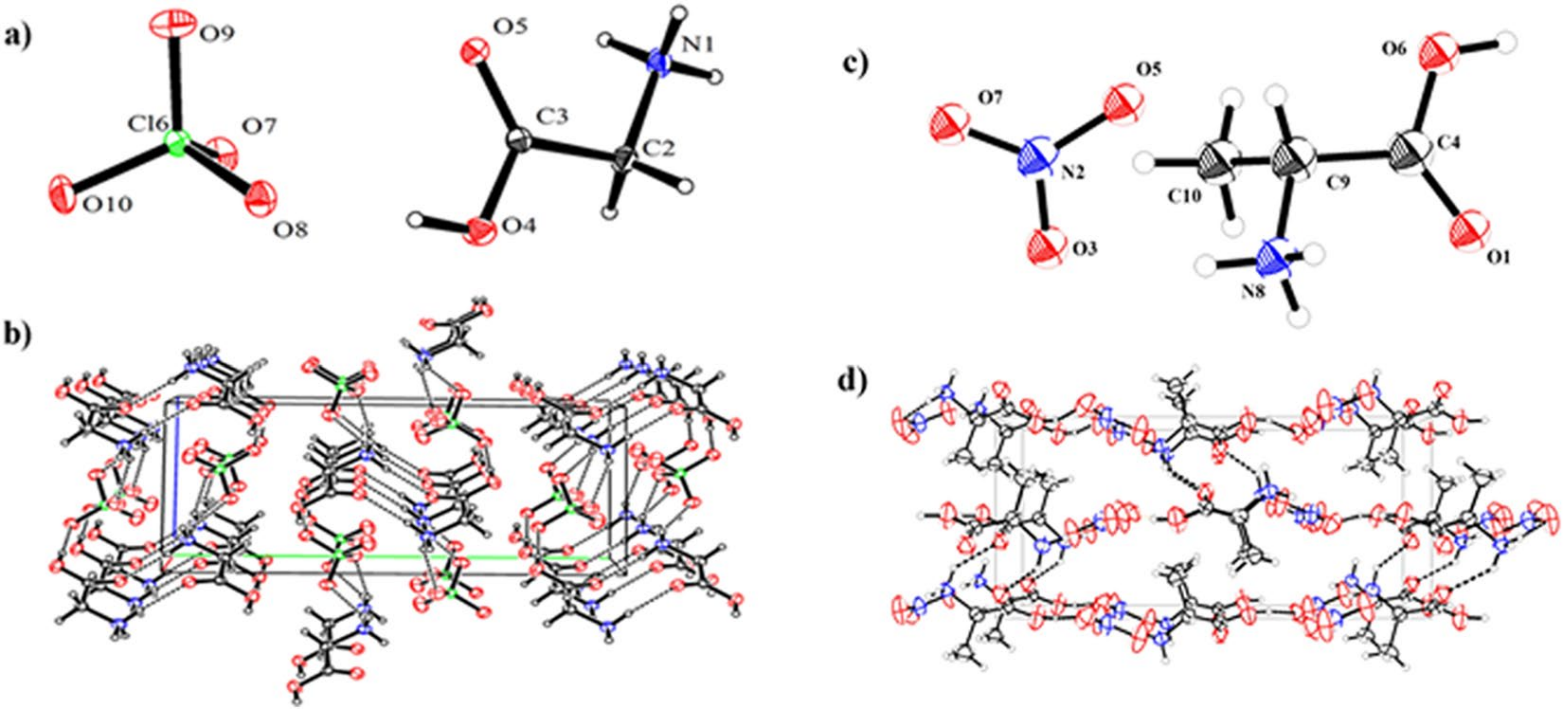
(**a**) Molecular structure of [Gly]ClO_4_. (**b**) Packing diagram of [Gly]ClO_4_ viewed down the *a*-axis. (**c**) Molecular structure of [Ala]NO_3_. (**d**) Packing diagram of [Ala]NO_3_ viewed down the *a*-axis. The unit cell is indicated, and the dashed lines represent hydrogen bonding. Thermal ellipsoids are drawn at the 50% probability level.

The hydrogen bond plays an important role in increasing the density and decreasing the sensitivity of energetic compounds^50,51^. The structures of [Gly]ClO_4_ and [Ala]NO_3_ were characterized by single-crystal X-ray diffraction (Fig. 5). [Gly]ClO_4_ crystallizes in the monoclinic space group *P*2_1_/*c* with four cation and four anion moieties in each unit cell. The structure is dominated by the interactions between cations and anions and hydrogen bonds (Fig. 5b). Each cation forms hydrogen bonds to three anions and another cation via the ammonium cation and carboxyl group. The donor-acceptor contact distances are in the range of 2.7422(18) to 2.9266(2) Å. The shortest H-acceptor distance starting from O(4) of the carboxyl group directly linked to O(8) of perchlorate is 2.7422(18) Å (O(4)−H(4)···O(8)). [Ala]NO_3_ crystallizes in the orthorhombic space group P2_1_2_1_2_1_ with four cation and four anion moieties in the unit cell. The packing structure of [Ala]NO_3_ is built up by hydrogen bonding and interactions between cations and anions along the *a* axis (Fig. 3d). Each NO_3_^−^ anion is surrounded by three alaninium cations oriented toward the oxygen atoms (O3, O5). The donor-acceptor contact distance range is 2.618(6) to 2.893(6) Å.

**Figure 6:**
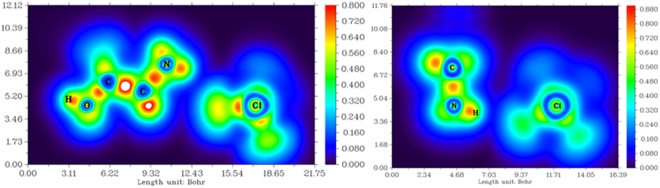
Color-filled map of localized orbital locator (LOL) of the cluster conformers in [Gly]ClO_4_.

**Figure 7:**
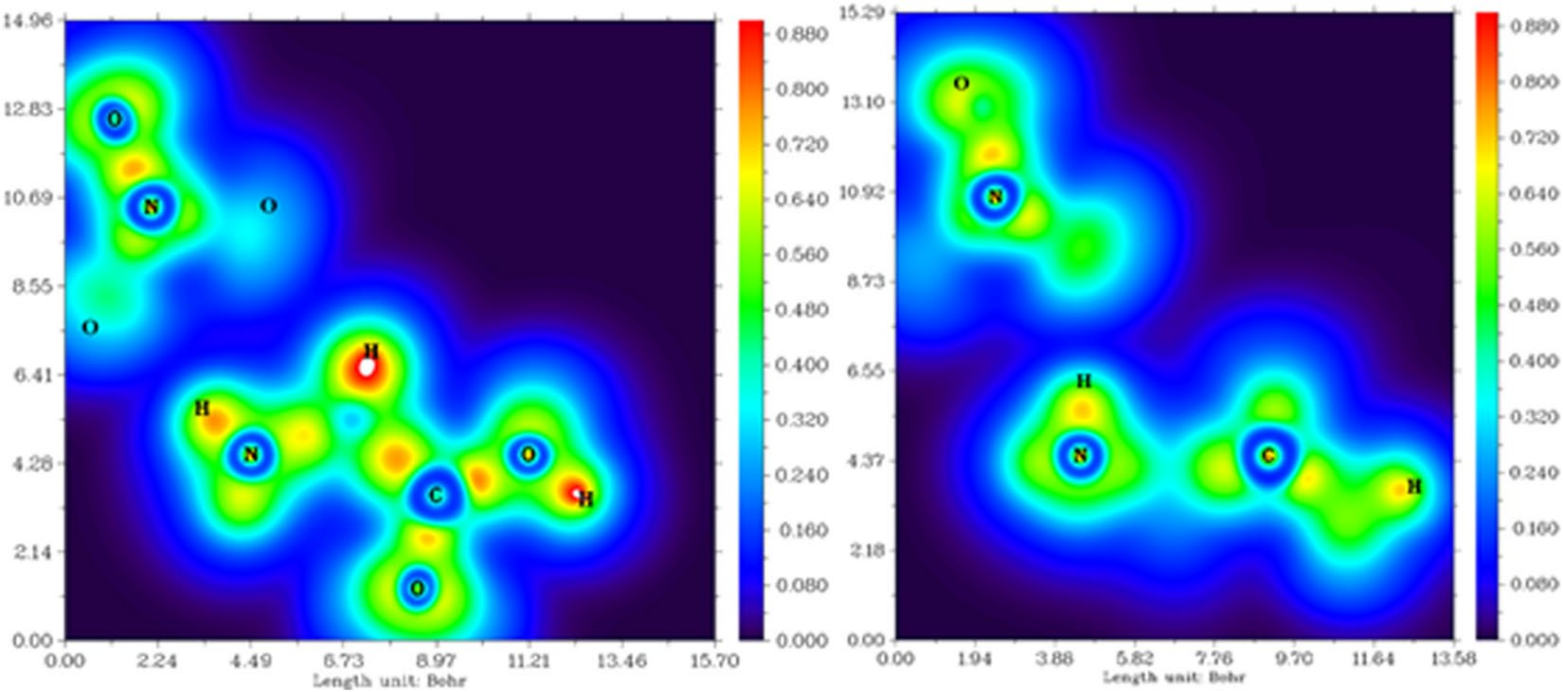
Color-filled map of localized orbital locator (LOL) of the cluster conformers in [Ala]NO_3_.

The localized orbital locator (LOL) that is dependent on the kinetic-energy density reveals the electronic shell structure of the compounds^52^. A large LOL value indicates that electrons are greatly localized, meaning that a covalent bond forms, with a lone pair or inner shells of an atom involved. The electron localization of [Gly]ClO_4_ and [Ala]NO_3_ cluster conformers where the NH_3_^+^ group connects directly to the ClO_4_^−^ anion are shown in Figs 6 and 7, respectively^53^. Covalent regions (e.g., C–C, C–N, and N–H) have high LOL values. The regions between the NH_3_^+^ group and ClO_4_^−^ anion have a low LOL value. indicating that [Gly]ClO_4_ is a classic ionic compound, and the low LOL value also implies that a hydrogen bond exists in the regions between the NH_3_^+^ group and ClO_4_^−^ anion^54,55^. The electron localization of [Ala]NO_3_ cluster conformers is similar to that of [Gly]ClO_4_ cluster conformers.

**Figure 8:**
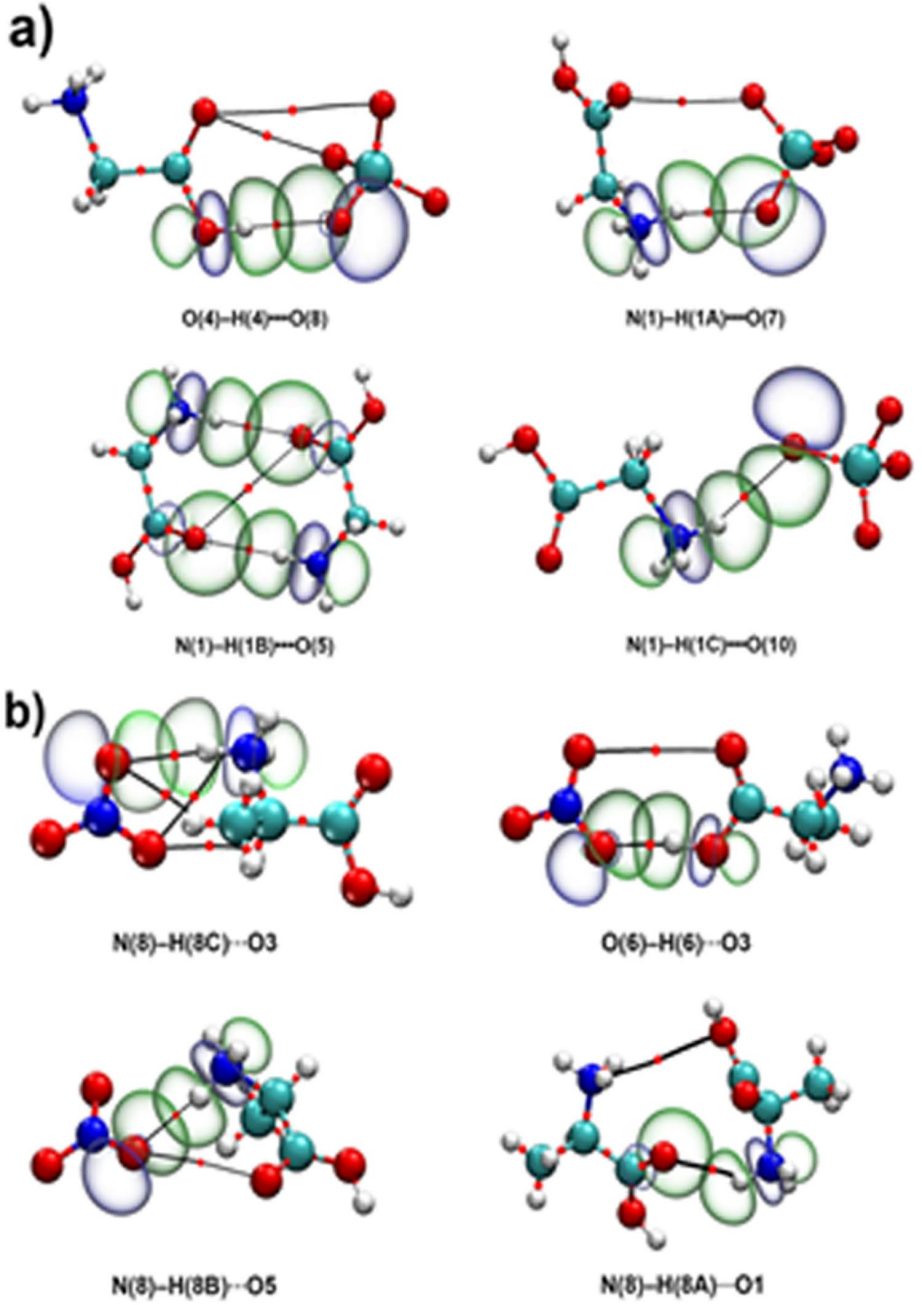
QTAIM and NBO analysis of the cluster conformers in [Gly]ClO_4_ (**a**) and [Ala]NO_3_ (**b**) crystal structure at the M062X/6–311 + + G(d,p) level. Larger spheres represent the atoms (C: grey, H: white, N: blue, O: red, Cl: light grey). Thick black lines indicate the bond paths of hydrogen bonds. The small red dots on the bond paths identify the BCPs. The natural bond orbitals isosurface is plotted at –0.05 a.u. and 0.05 a.u., respectively.

To explain the H-bonding, the quantum theory of atoms in molecules (QTAIM) analysis was used^56–58^. The topological criteria for the existence of hydrogen bonding is the bond path between the hydrogen atom and acceptor with the bond critical point (BCP) occurring at the minimum electron density *ρ*(r) (*ρ*_BCP_) and an appropriate gradient, the Laplacian of the electron density ∇^2^ *ρ*(r) (∇^2^_*ρ*BCP_) (Fig. 8). Table 2 lists the H-bonds as determined by the existence of a BCP and identifies these as weak and medium H-bonds in the [Gly]ClO_4_ crystal. The O4–H4–O8 bond with *ρ*_BCP_ = 0.0247 e bohr^−3^ (∇^2^_ρBCP_ = 0.1074 e bohr^−5^) lies well within the medium H-bonding range. These hydrogen bonds in the crystal might contribute to closer packing to obtain the high density of [Gly]ClO_4_. Table 3 lists the H-bonds as determined by the existence of a BCP and identifies these as medium and strong H-bonds in the [Ala]NO_3_ crystal. The O6–H6–O3 bond with *ρ*_BCP_ = 0.0424 e bohr^−3^ (∇^2^_ρBCP_ = 0.1323 e bohr^−5^) lies well within the strong H-bonding range. The natural bond orbital (NBO) analysis is also a key approach to the study of H-bonding^57,58^. The interactions between the empty acceptor-hydrogen (A–H) antibonding orbital (σ^*^) and the filled lone pair orbital (n) on the donor (D) are shown in Fig. 8. The orbital overlap represents the charge transfer from the occupied donor lone-pair orbital into the empty antibonding orbital. The stabilization energy E^(2)^_(n→σ*)_ associated with the amount of electron density donated from the occupied donor lone-pair orbital to the empty antibonding orbital is obtained. The values for E^(2)^_(n→σ*)_ in [Gly]ClO_4_ range from 10.71 to 30.19 kJ mol^−1^ (Table 3). The shortest hydrogen bond (O4–H4–O8) has the highest value of 30.19 kJ mol^−1^ in the [Gly]ClO_4_ crystal, indicating medium H- bonding, which is in agreement with the QTAIM analysis. The E^(2)^_(n→σ*)_ value in [Ala]NO_3_ is 9.07 to 87.70 kJ mol^−1^, whereas that of O6–H6–O3 bond is the highest (87.80 kJ mol^−1^). In [Gly]ClO_4_, each anion forms hydrogen bonds to three glycinium cations with E^(2)^ 30.19, 10.71 and 13.72 kJ mol^−1^, respectively, which might help to stabilize the irascible ClO_4_^−^ and decrease the sensitivity of [Gly]ClO_4_. A similar situation is also present in [Ala]NO_3_.

**Table 2:** Hydrogen bonds in [Gly]ClO_4_ crystal.

D	H	A	d(D-H)/Å	d(H-A)/Å	d(D-A)/Å	<(DHA)/°	*ρ*_BCP_/e Å^−3^	∇^2^_ρBCP_/e Å^−5^	E(2)/kJ mol^−1^	Strength
O4	H4	O8	0.82	1.92	2.742 (2)	177.0	0.0247	0.1074	30.19	Medium
N1	H1A	O7^1^	0.89	2.16	2.922(2)	143.6	0.0164	0.0642	10.71	Weak
N1	H1B	O5^2^	0.89	2.04	2.926 (2)	175.5	0.0176	0.0807	12.04	Weak
N1	H1C	O10^3^	0.89	2.11	2.870(2)	143.2	0.0180	0.0732	13.72	Weak

^1^1 + X, + Y, −1 + Z; ^2^1−X, 1−Y, 1−Z; ^3^1 + X, + Y, −1 + Z.

**Table 3:** Hydrogen bonds in [Ala]NO_3_ crystal.

D	H	A	d(D-H)/Å	d(H-A)/Å	d(D-A)/Å	<(DHA)/°	*ρ*_BCP_/e bohr^−3^	∇^2^_ρBCP_/e bohr^−5^	E(2)/kJ mol^−1^	Strength
N8	H8C	O3	0.89	2.02	2.893(6)	167.7	0.0228	0.0857	34.67	Medium
O6	H6	O3^1^	0.93	1.70	2.618(6)	167.0	0.0424	0.1323	87.70	Strong
N8	H8B	O5^2^	0.89	1.96	2.831(6)	165.7	0.0257	0.0967	40.48	Medium
N8	H8A	O1^3^	0.89	2.14	2.876(5)	138.9	0.0141	0.0792	9.07	Weak

^1^1/2 − X, −1 − Y, 1/2 + Z; ^2^ −1 + X, + Y, + Z; ^3^ 1/2 + X, −1/2 − Y, −1 − Z.

**Figure 9:**
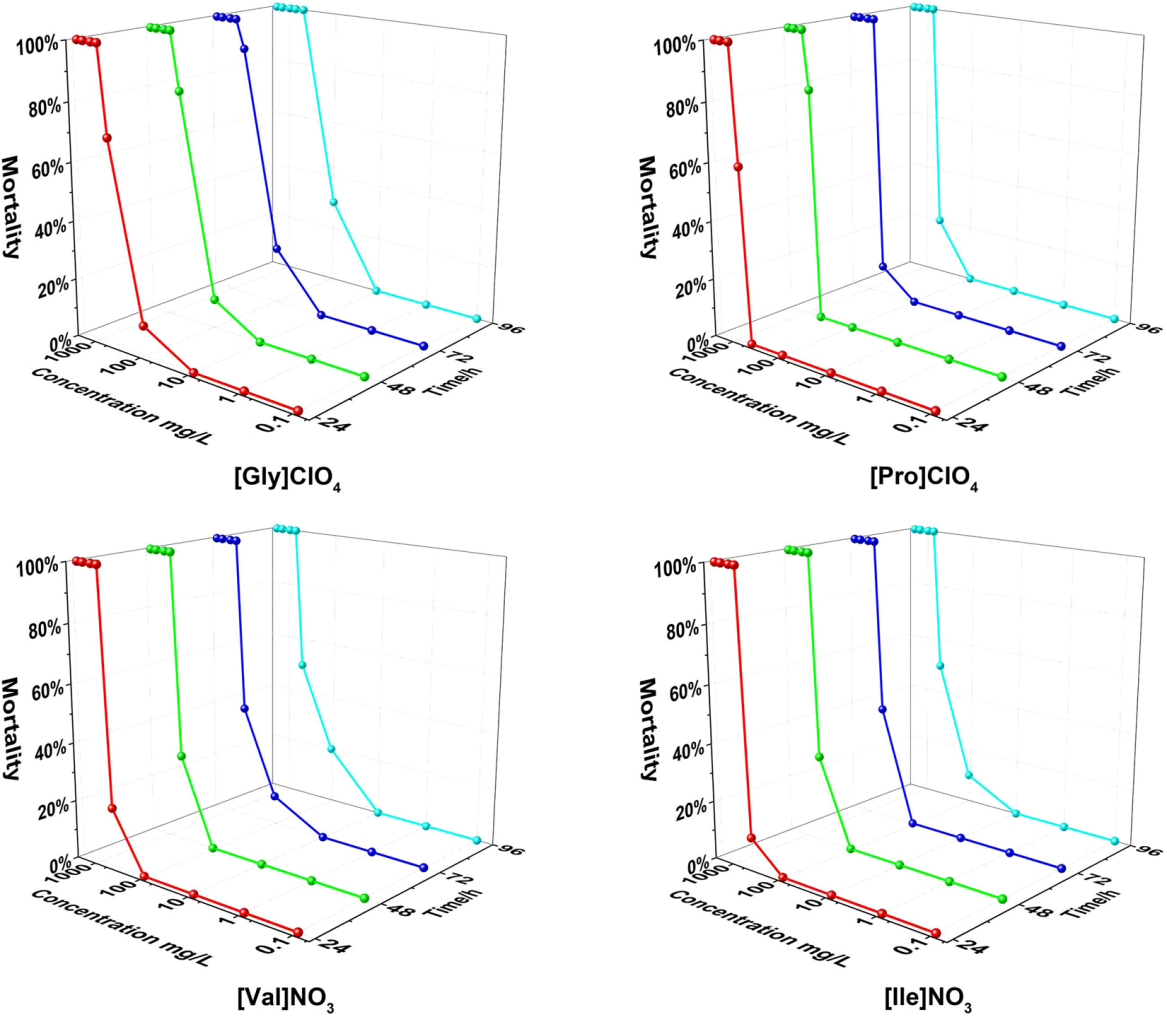
IBEM acute lethality curves of Macrobrachium nipponense determined at different IBEM concentrations in the exposed environment.

The toxicity of energetic materials is an important concern for the public. To assess the acute lethal toxicity of IBEMs on aquatic animals, diluted aqueous solutions of selected high-quality energetic amino acid salts/ionic liquids were administered to the model species Macrobrachium nipponense. Macrobrachium nipponense is a dominant species in the freshwater and stream ecosystems and is a sensitive bio-indicator of pollutants^59^. The acute lethality curves of [Gly]ClO_4_, [Pro]ClO_4_, [Val]NO_3_ and [Ile]NO_3_ with respect to Macrobrachium nipponense are shown in Fig. 9. The mortality increases with the initial concentration of IBEMs. The median lethal concentrations (*LC*_50_), the most important toxicological parameter, were calculated according to the lethal rates of Macrobrachium nipponense. The value of *LC*_50_ for an examined compound is the dose required to kill half of the members of a tested population after a specified test period. It is obvious that the longer the test duration, the shorter are the median lethal concentrations (24 h, 48 h, 72 h and 96 h *LC*_50_) of the four IBEMs to Macrobrachium nipponense, as presented in Table 4. The 96 h *LC*_50_ values for [Gly]ClO_4_, [Pro]ClO_4_, [Val]NO_3_ and [Ile]NO_3_ to Macrobrachium nipponense are 367.65 (271.40–484.66), 648.80 (527.53–760.12), 471.86 (367.02–603.80) and 523.02 (412.76–647.49) mg L^−1^, which exceed the low toxicity critical value of 100 mgL^−160^. These IBEMs are apparently hypotoxic to Macrobrachium nipponense in the acute lethal toxicity assay, although in large amounts, perchlorate interferes with iodine uptake into the thyroid gland^61,62^.

**Table 4:** Median lethal concentrations (*LC*_50_) to Macrobrachium nipponense.

*LC*_50_ mg L^−1^				
	24 h	48 h	72 h	96 h
[Gly]ClO_4_	425.89	398.40	398.40	367.65
	(324.57–543.18)	(299.53– 514.83)	(299.53–514.83)	(271.40–484.66)
[Pro]ClO_4_	726.82	703.25	674.62	648.80
	(514.15–869.64)	(529.98–795.06)	(572.86–779.92)	(527.53–760.12)
[Val]NO_3_	563.55	563.55	520.20	471.86
	(498.68–1786.20)	(498.68–1786.20)	(410.17–645.65)	(367.02–603.80)
[Ile]NO_3_	592.43	592.43	547.51	523.02
	(520.49–725.99)	(520.49–725.99)	(356.99–778.84)	(412.76–647.49)

^a^The 95% confidence limits are given in parentheses.

## Conclusion

In conclusion, sixteen amino acid salts/ionic liquids, including [AA]ClO_4_ and [AA]NO_3_ materials, were systematically studied as ionic bio-energetic materials (IBEMs). We present a new approach for the development of bio-energetic materials. [AA]ClO_4_ and [AA]NO_3_ were easily prepared from bio-renewable amino acids and possessed high densities as well as low sensitivities. The combustion of these compounds is more intense than that of certain conventional NEMs. The energetic properties of several IBEMs are comparable to those of TNT, TNP, RDX and HMX. These IBEMs preserve the benign environment from the natural source and display excellent hypotoxicity. Our work suggests that IBEMs are promising candidates for bio-based energetic materials.

## Experimental Section

### General Methods

All chemicals were obtained commercially as analytical-grade materials and used as received. Solvents were dried using standard procedures. The ionic bio-energetic materials [AA]X were synthesized by direct acidification from amino acids and corresponding acids in water for 24 h and subsequently dried by evaporation of water in air followed by vacuum drying, as described in our previous studies^38^. Infrared spectra (IR) were recorded on a NEXUS 670 FT-IR spectrometer on KBr pellets. ^1^H and ^13^C NMR spectra were recorded on a Bruker 400 MHz nuclear magnetic resonance spectrometer operating at 400 and 100 MHz, respectively, with *d*_6_-DMSO as the locking solvent. The ^1^H and ^13^C chemical shifts are reported in ppm relative to TMS. Coupling constants are given in Hertz. Elemental analyses (H, C, N) were performed on an Elementar Vario MICRO CUBE elemental analyzer. Decomposition temperatures were characterized using a thermogravimetric analyzer (TGA) on a NETZSCH TG 209F1 calorimeter. The heat flow of these materials was obtained by differential thermal analysis (DTA) using a NETZSCH TG 209F1 calorimeter. Measurements were accomplished by heating the samples at a heating rate of 10 °C min^−1^ from 25 to 600 °C. Melting points were determined by differential scanning calorimetry (DSC) on a TA Q20 calorimeter calibrated with standard pure indium. Measurements were performed at a heating rate of 10 °C min^−1^ with a nitrogen flow rate of 20 mL min^−1^. The reference sample was an Al container with nitrogen. The densities were measured at 25 °C using a pycnometer. The experimental enthalpy of combustion was measured using a Parr 6725 bomb calorimeter (static jacket) equipped with a Parr 207 A oxygen bomb. Initial safety testing of amino acids salts/ionic liquids with respect to impact and friction were performed using the BAM method. The sensitivity towards impact (IS) was tested by the action of a falling weight from different heights. The friction sensitivity (FS) was determined by rubbing a small amount of material between a porcelain plate and a pin with different contact pressures. Macrobrachium nipponense were obtained from local commercial suppliers and transported to a plastic aquarium equipped with an air pump to aerate the water. The temperature was maintained at 20.0 ± 0.5 °C. Macrobrachium nipponense were acclimated for one week and those with body length 5 ± 0.5 cm were used in acute toxicity tests in the initial experiments. Stock solutions were prepared in deionized water. Laboratory tests were conducted to determine the median lethal concentration (*LC*_50_) for Macrobrachium nipponense. Ten animals were randomly sampled and placed in plastic beakers. After 24 h acclimatization, Macrobrachium nipponense were exposed to different concentrations of [Gly]ClO_4_, [Pro]ClO_4_, [Val]NO_3_ and [Ile]NO_3_ for 96 h. During the experiment, dead animals were removed over time. Mortality was recorded after 24, 48, 72 and 96 h. The *LC*_50_ values of every test chemical with 95% confidence limits were calculated for Macrobrachium nipponense using the probit analysis^63^.

### X-ray Crystallography

Single crystals of [Gly]ClO_4_ were removed from the flask, and a suitable crystal was selected and attached to a glass fiber. The data were collected by a New Gemini, Dual, EosS2 diffractometer with graphite-monochromated Mo-Kα radiation (*λ* = 0.71073 Å). Single crystals of [Ala]NO_3_ were removed from the flask, and a suitable crystal was selected and attached to a glass fiber. The data were collected by a New Gemini, Dual, EosS2 diffractometer with graphite-monochromated Cu-Kα radiation (*λ* = 1.54184 Å). The crystal was held at 293 K during data collection. Using Olex2^64^, the structure was solved with the ShelXT^65^ structure solution program using Direct Methods and refined with the XL^66^ refinement package using least squares minimization. All non-hydrogen atoms were refined anisotropically, and hydrogen atoms were located and refined. No decomposition was observed during data collection. Crystal data and structure refinement for [Gly]ClO_4_ and [Ala]NO_3_ are given in Table 5. Details of the data are given in Tables S1–S4.

**Table 5:** Crystal data and structure refinement for [Gly]ClO_4_ and [Ala]NO_3_.

Crystal	[Gly]ClO_4_	[Ala]NO_3_
Empirical formula	C2H6ClNO6	C3H7N2O5
Formula weight/g mol^−1^	175.53	151.11
T/K	293	293
Crystal system	Monoclinic	Orthorhombic
Space group	P2_1_/c	P2_1_2_1_2_1_
a/Å	5.1759(2)	5.6597(3)
b/Å	16.2326(5)	7.4660(5)
c/Å	7.5140(3)	16.1333(9)
α	90	90
β	101.868(4)	90
γ	90	90
V/Å^3^	617.82(4)	681.72(7)
Z	4	4
ρ/g cm^–3^	1.887	1.472
μ/mm^–1^	0.595	1.256
F(000)	360.0	316.0
*λ*Kα/Ǻ	0.71073 (Mo)	1.54184 (Cu)
Reflns	7843	2364
Rint Independent reflections	0.0309	0.0604
S on F^2^	1.172	1.133
Params	93	98
*R*_1_ (*I* > 2*σ* (I))^a^	0.0382	0.0778
w*R*_2_ (*I* > 2*σ* (I))^b^	0.0946	0.2187
*R*_1_ (all data)^a^	0.0422	0.0830
w*R*_2_ (all data)^b^	0.0969	0.2296

### Theoretical Study

Computations for the heat of combustion were performed using the Gaussian09 (Revision A.02) suite of programs^47^. The geometric optimization and frequency analyses were performed using Møller-Plesset second-order perturbation theory truncated at the second order (MP2) with the 6–311++G** basis set^67,68^. All of the optimized structures were characterized as true local energy minima on the potential energy surface without imaginary frequencies. The theoretical heats of combustion were simplified using the expression:

1 $${{\rm{\Delta }}}_{{\rm{c}}}H^\circ ([{\rm{AA}}]X,\,298\,{\rm{K}})={{\rm{\Sigma }}{\rm{\Delta }}}_{{\rm{f}}}H^\circ \,(\text{product},\,\text{298}.15\,{\rm{K}})-{{\rm{\Sigma }}{\rm{\Delta }}}_{{\rm{f}}}H^\circ (\text{reaction},\,\text{298}.15\,{\rm{K}})$$

The theoretical heats of formation of the cations and anions were computed using the method of protonation reactions (Figure S1). The sources of the energies of the parent ions in the isodesmic reactions were calculated from protonation reactions Δ_f_ *H*° (H^+^) = +1528.085 kJ mol^−1^ ^69^. The enthalpies of reaction (Δ_r_ *H*°, 298.15) were obtained by combining the MP2/6–311++G** energy differences for the reaction, the scaled zero point energies, and other thermal factors. The theoretical heats of formation of the [AA]ClO_4_ and [AA]NO_3_ at T = 298.15 K were calculated based on a Born-Haber energy cycle (Figure S2). In a Born-Haber energy cycle, the heat of formation of an AAIL can be simplified by the following expression:

2 $${{\rm{\Delta }}}_{{\rm{f}}}H^\circ ([{\rm{AA}}]{\rm{X}},\,298\,{\rm{K}})={{\rm{\Sigma }}{\rm{\Delta }}}_{{\rm{f}}}H^\circ \,({\rm{cation}},\,298.15\,{\rm{K}})\,+\,{{\rm{\Sigma }}{\rm{\Delta }}}_{{\rm{f}}}H^\circ \,({\rm{anion}},\,298.15\,{\rm{K}})\,-\,{{\rm{\Delta }}}_{{\rm{f}}}{H}_{{\rm{L}}}$$

where Δ_f_ *H*_L_ is the lattice energy of the ionic salt, and Δ_f_ *H*_L_ (kJ mol^−1^) can be predicted by the formula suggested by Jenkins *et al*.^70^:

3 $${{\rm{\Delta }}}_{{\rm{f}}}{H}_{{\rm{L}}}={U}_{{\rm{POT}}}+[p({{\rm{n}}}_{{\rm{M}}}/2\,-\,2)+{\rm{q}}({{\rm{n}}}_{{\rm{X}}}/2\,-\,2)]RT$$

where n_M_ and n_X_ depend on the nature of the ions M^p+^ and X^q−^, respectively, and have a value of six for nonlinear polyatomic ions. The equation for lattice potential energy *U*_POT_ has the form:

4 $${U}_{{\rm{POT}}}=\gamma {({\rho }_{{\rm{m}}}{/{\rm{M}}}_{{\rm{m}}})}^{1/3}+\delta $$

where *ρ*_m_ is the density (g cm^−3^) and M_m_ is the chemical formula mass of the [AA]ClO_4_ and [AA]NO_3_. For MX (1:1) salts, *γ* is 1981.2 and *δ* is 103.8. Thus, *U*_POT_ of [AA]ClO_4_ and [AA]NO_3_ is given by:

5 $${U}_{{\rm{POT}}}=1981.2{({\rho }_{{\rm{m}}}{/{\rm{M}}}_{{\rm{m}}})}^{1/3}+103.8$$

The LOL, QTAIM and NBO analysis of the cluster conformers in the [Gly]ClO_4_ crystal structure at the M062X/6–311++G (d, p) level was performed by the Gaussian09 (Revision A.02) suite of programs^47^. The Gaussian output wfn files were used as inputs for Multiwfn to perform the QTAIM analysis^53^. The LOL analysis map was drawn by Multiwfn. The AIM topological analysis diagram was drawn by Multiwfn and VMD^71^. The NBO was plotted by Multiwfn and VMD using the Gaussian output fch files.

### Detonation property

The detonation parameters were calculated using the Kamlet-Jacobs equation^72^:

6 $$D=(1.01+1.312{\rho }_{{\rm{m}}}){{\rm{\varphi }}}^{1/2}$$

7 $$P=1.558{{\rho }_{{\rm{m}}}}^{2}{{\rm{\varphi }}}^{1/2}$$

8 $${\rm{\varphi }}={{\rm{NM}}}^{1/2}{{\rm{Q}}}^{1/2}$$

where *P* is the detonation pressure (GPa), *D* is the detonation velocity (km s^−1^), *ρ*_m_ is the packed density (g cm^−3^), ϕ is the characteristic value of explosives, N is the moles of gas produced per gram of explosives (mol g^−1^), M is an average molar weight of detonation products (g mol^−1^), and Q is the maximum estimation heat of detonation (cal g^−1^).

## Electronic supplementary material

Supplementary information
