# Supplementary material for: Insensitive ionic bio-energetic materials derived from amino acids
Source: Sci Rep. 2017 Oct 6;7:12744. doi: 10.1038/s41598-017-12812-7 (PMC5630594; doi:10.1038/s41598-017-12812-7)
Supplement: Supplementary file 1 — Supplementary information [file 41598_2017_12812_MOESM1_ESM.doc]

Supplementary information

Insensitive ionic bio-energetic materials derived from amino acids

Lei Zhang, Kang-Xiang Song, Zhang Zhang, Wen-Li Yuan, Nanrong Zhao, Song Qin, Ling He* and Guo-Hong Tao*

College of Chemistry, Sichuan University, Chengdu 610064, China.

E-mail address: [lhe@scu.edu.cn](mailto:lhe@scu.edu.cn), taogh@scu.edu.cn

**Index:**

**S2-S6 Experimental**

**S7 Figure S1 Protonation Reactions of Ions in Amino Acid Salts**

**S8 Figure S2 Born-Haber Cycle for Heats of Formation**

**S8 Figure S3 Combustion Reaction Equations**

**S9 Figure S4 Thermogravimetry analysis (TGA) Curves of Amino Acid Salts**

**S9 Figure S5 Differential thermal analysis (DTA) Curves of Amino Acid Salts**

**S10-S11 Figure S6-S13 Selected frames of the combustion of Amino Acid Salts**

**S12-S13 Table S1-S4 Crystallography Data of [Gly]ClO_4_ and [Ala]NO_3_**

**S14-S15 The geometries of the four cluster conformers in crystal [Gly]ClO_4_**

**S16-S17 The geometries of the four cluster conformers in crystal [Ala]NO_3_**

**S19-****S21 The optimized geometry coordinates of 8 amino acid cations**

**S21-S23 The optimized geometry coordinates of 8 amino acids**

**S21-S23 The optimized geometry coordinates of 8 amino acids**

**Experimental**

**Materials**

Glycine (99.5%), *L*-alanine (99%), *L*-valine (99%), *L*-leucine (99%) and *L*-proline (99%) were purchased from Sinopharm Chemical Reagent Co., Ltd (China). *L*-Sercine (98%), *L*-aspartic acid (99%) and *L*-isoleucine (99%) were purchased from J&K Scientific Ltd. (China). Perchloric acid (HClO_4_, 70%) and nitric acid (HNO_3_, 65%) were purchased from Chengdu Kelong Chemical Reagent Factory (China). All reagents were used as received.

**Glycine perchlorate ([Gly]ClO_4_).** In a 50 mL flask, equivalent mole of perchloric acid solution (70%) was added into 20 mL of glycine (0.751 g, 10 mmol) solution. The resulting mixture was stirred at room temperature for 24 h. Then the mixture was dried to yield [Gly]ClO_4_ (1.502 g, 99%) as a colorless solid. IR (KBr, 25 °C): *ν* = 3425 (s), 3004 (s), 1747 (vs), 1628 (m), 1496 (m), 1423 (m), 1247 (m), 1142 (s), 1113 (s), 1085 (s), 630(s) cm^–1^. ^1^H NMR (400 MHz, *d*_6_-DMSO): *δ* = 8.04 (s, 3H, NH_3_), 3.69 (s, 2H, CH_2_) ppm. ^13^C NMR (100 MHz, *d*_6_-DMSO): *δ* = 169.12, 39.78 ppm. Elemental analysis calcd (%) for C2H6NO6Cl (175.52): C 13.69, H 3.45, N 7.98; found C 13.56, H 3.39, N 7.65.

**Alanine perchlorate ([Ala]ClO_4_).** The similar procedure was followed as that described above for the preparation of [Gly]ClO_4_**.** Alanine (0.892 g, 10 mmol) and equivalent mole of perchloric acid solution (70%) were reacted in deionized water to give [Ala]ClO_4_ as a white solid (1.659 g, >99%). IR (KBr, 25 °C): *ν* = 3432 (s), 2993 (m), 1726 (vs), 1624 (m), 1492 (s), 1389 (w), 1200 (s), 1108 (vs), 816 (m), 631 (s) cm^–1^. ^1^H NMR (400 MHz, *d*_6_-DMSO): *δ* = 8.12 (s, 3H, CH_3_), 3.95 (q, *J* = 7.2 Hz, 1H, CH), 1.36 (d, *J* = 7.2 Hz, 3H, CH_3_) ppm. ^13^C NMR (100 MHz, *d*_6_-DMSO): *δ* = 171.60, 47.88, 15.75 ppm.

**Serinium perchlorate** (**[Ser]ClO_4_**). The similar procedure was followed as that described above for the preparation of [Gly]ClO_4_. Serine (1.051 g, 10 mmol) and equivalent mole of perchloric acid solution (70%) were reacted in deionized water to give [Ser]ClO_4_ as a white solid (1.758 g, 96%). IR (KBr, 25 °C): *ν* = 3425 (s), 2964 (m), 1738 (vs), 1624 (m), 1500 (s), 1259 (w), 1140 (s), 1113 (s), 1085 (vs), 632 (s) cm^–1^. ^1^H NMR (400 MHz, *d*_6_-DMSO): *δ* = 13.78 (s, 1H, COOH), 8.18 (s, 3H, NH_3_), 5.53 (s, 1H, OH), 4.00 (s, 1H, CH), 3.78 (ddd, *J* = 14.7, 11.5, 3.8 Hz, 2H) ppm. ^13^C NMR (100 MHz, *d*_6_-DMSO): *δ* = 169.56, 59.42, 53.20 ppm. Elemental analysis calcd (%) for C3H8NO7Cl (175.52): C 17.53, H 3.92, N 6.81; found C 17.64, H 4.32, N 6.78.

**Aspartate perchlorate** (**[Asp]ClO_4_**). The similar procedure was followed as that described above for the preparation of [Gly]ClO_4_. Aspartic acid (1.334 g, 10 mmol) and equivalent mole of perchloric acid solution (70%) were reacted in deionized water to give [Asp]ClO_4_ as a white solid (2.073 g, 99%). IR (KBr, 25 °C): *ν* = 3541 (s), 3220 (s), 2964 (m), 1742 (vs), 1610 (m), 1494 (s), 1402 (m), 1213 (w), 1091 (s), 625 (s) cm^–1^. ^1^H NMR (400 MHz, *d*_6_-DMSO): *δ* = 13.05 (s, 1H, COOH), 8.22 (s, 3H, NH_3_), 4.17 (s, 1H, CH), 2.88–2.74 (m, 2H, CH_2_) ppm. ^13^C NMR (100 MHz, *d*_6_-DMSO): *δ* = 171.07, 170.08, 48.60, 34.40 ppm.

**Valnium perchlotate ([Val]ClO_4_).** The similar procedure was followed as that described above for the preparation of [Gly]ClO_4_. Valine acid (1.172 g, 10 mmol) and equivalent mole of perchloric acid solution (70%) were reacted in deionized water to give [Val]ClO_4_ as a white solid (1.842 g, 95%). IR (KBr, 25 °C): *ν* = 3438 (s), 3010 (s), 2912 (m), 1728 (vs), 1481 (s), 1209 (s), 1146 (s), 1115 (w), 1095 (s), 631 (s) cm^–1^. ^1^H NMR (400 MHz, *d*_6_-DMSO): *δ* = 8.12 (s, 3H, NH_3_), 3.82–3.74 (m, 1H, CH), 2.20–2.09 (m, 1H, CH), 0.97 (dd, J = 7.0, 3.1 Hz, 6H, CH_3_, CH_3_) ppm. ^13^C NMR (100 MHz, *d*_6_-DMSO): *δ* = 171.42, 58.33, 30.06, 18.93, 18.73 ppm.

**Isoleucinium perchlorate ([Ile]ClO_4_).** The similar procedure was followed as that described above for the preparation of [Gly]ClO_4_. Isoleucine (1.311 g, 10 mmol) and equivalent mole of perchloric acid solution (70%) were reacted in deionized water to give [Ile]ClO_4_ as a white solid (2.060 g, 99%). IR (KBr, 25 °C): *ν* = 3433 (s), 3113 (m), 2964 (s), 1720 (vs), 1623 (m), 1483 (m), 1400 (m), 1213 (w), 1146 (s), 1113 (s), 1086 (vs), 806 (m), 631 (s) cm^–1^. ^1^H NMR (400 MHz, *d*_6_-DMSO): *δ* = 8.14 (s, 3H, NH_3_), 3.86 (d, *J* = 3.2 Hz, 1H, CH), 2.00–1.65 (m, 1H, CH), 1.47 (ddd, *J* = 13.3, 7.3, 5.7 Hz, 1H, CH_2_), 1.35–1.22 (m, 1H, CH_2_), 0.98–0.85 (m, 6H, CH_3_, CH_3_) ppm. ^13^C NMR (100 MHz, *d*_6_-DMSO): *δ* = 170.44, 56.26, 35.84, 25.16, 14.50, 11.68 ppm. Elemental analysis calcd (%) for C6H14NO6Cl (175.52): C 31.11, H 6.09, N 6.05; found C 30.91, H 6.04, N 5.86.

**Leucinium perchlorate ([Leu]ClO_4_, 7).** The similar procedure was followed as that described above for the preparation of [Gly]ClO_4_. Leucine (1.311 g, 10 mmol) and equivalent mole of perchloric acid solution (70%) were reacted in deionized water to give [Leu]ClO_4_ as a white solid (2.061 g, 99%). IR (KBr, 25 °C): *ν* = 3548 (m), 3158 (m), 2965 (s), 2877 (s), 1744 (vs), 1602 (m), 1576 (m), 1480 (m), 1238 (w), 1206 (vs), 1066 (s), 975 (s), 929 (m), 802 (m), 619 (s), 525 (s) cm^–1^. ^1^H NMR (400 MHz, DMSO) δ 13.82 (s, 1H), 8.15 (s, 3H), 3.87 (s, 1H), 1.85–1.49 (m, 3H), 0.90 (dd, *J* = 6.5, 2.8 Hz, 6H). 1.47 (ddd, *J* = 13.3, 7.3, 5.7 Hz, 1H, CH_2_), 1.35–1.22 (m, 1H, CH_2_), 0.98–0.85 (m, 6H, CH_3_, CH_3_) ppm. ^13^C NMR (100 MHz, DMSO) δ 171.66 (s), 50.60 (s), 23.83 (s), 22.31 (s), 21.93 (s). Elemental analysis calcd (%) for C6H14NO6Cl (175.52): C 31.11, H 6.09, N 6.05; found C 30.85, H 5.99, N 5.87.

**Prolinium perchlorate ([Pro]ClO_4_).** The similar procedure was followed as that described above for the preparation of [Gly]ClO_4_. Proline (1.157 g, 10 mmol) and equivalent mole of perchloric acid solution (70%) were reacted in deionized water to give [Pro]ClO_4_ as a white solid (1.843 g, 96%). IR (KBr, 25 °C): *ν* = 3396 (s), 3006 (s), 1745 (vs), 1556 (w), 1429 (s), 1365 (s), 1196 (s), 1101 (vs), 939 (m), 843 (m), 766 (s), 627 (s) cm^–1^. ^1^H NMR (400 MHz, *d*_6_-DMSO): *δ* = 9.26 (s, 1H, CH_2_), 8.67 (s, 1H, CH_2_), 4.71–3.96 (m, 1H, CH), 3.57–2.89 (m, 2H, CH_2_), 2.40–2.14 (m, 1H, CH_2_), 1.93 (ddd, *J* = 18.9, 13.9, 7.2 Hz, 3H, CH_2_, CH_2_) ppm. ^13^C NMR (100 MHz, *d*_6_-DMSO): *δ* = 170.47, 58.83, 45.50, 27.76, 23.07 ppm. Elemental analysis calcd (%) for C5H10NO6Cl (215.59): C 27.86, H 4.68, N 6.50; found C 27.50, H 4.81, N 6.29.

**Glycinium nitrate ([Gly]NO_3_).** In a 50 mL flask, equivalent mole of nitric acid solution (65%) was added into 20 mL of glycine (0.751 g, 10 mmol) solution. The resulting mixture was stirred at room temperature for 24 h. Then the mixture was dried to yield [Gly]NO_3_ (1.380 g, >99%) as a white solid. IR (KBr, 25 °C): *ν* = 3406 (m), 3220 (s), 3006 (s), 1753 (s), 1529 (m), 1417 (w), 1386 (vs), 1332 (m), 1234 (s), 1120 (m), 1039 (m), 908 (m), 872 (m) cm^–1^. ^1^H NMR (400 MHz, *d*_6_-DMSO): *δ* = 8.14 (s, 3H, NH_3_), 3.69 (s, 2H, CH_2_) ppm. ^13^C NMR (100 MHz, *d*_6_-DMSO): *δ* = 169.10, 39.94 ppm.

**Alaninium nitrate ([Ala]NO_3_).** The similar procedure was followed as that described above for the preparation of [Gly]NO_3_. Alanine (0.890 g, 10 mmol) and equivalent mole of nitric acid solution (65%) were reacted in deionized water to give [Ala]NO_3_ (1.453 g, 96%) as a white solid. IR (KBr, 25 °C): *ν* = 3211 (s), 3014 (s), 2960 (s), 1726 (s), 1518 (m), 1410 (s), 1385 (vs), 1334 (s), 1221 (s), 1201 (s), 1144 (m), 1111 (m), 867 (m), 814 (s) cm^–1^. ^1^H NMR (400 MHz, *d*_6_-DMSO): *δ* = 8.23 (s, 3H, NH_3_), 3.94 (q, *J* = 7.2 Hz, 1H, CH), 1.37 (d, *J* = 7.2 Hz, 3H, CH_3_) ppm. ^13^C NMR (100 MHz, *d*_6_-DMSO): *δ* = 171.69, 48.13, 15.93 ppm.

**Serinium nitrate ([Ser]NO_3_).** The similar procedure was followed as that described above for the preparation of [Gly]NO_3_. Serine (1.050 g, 10 mmol) and equivalent mole of nitric acid solution (65%) were reacted in deionized water to give [Ser]NO_3_ (1.621 g, 96%) as a colorless viscous liquid. IR (KBr, 25 °C): *ν* = 3394 (s), 3076 (s), 1734 (s), 1593 (m), 1496 (s), 1390 (vs), 1234 (s), 1130 (m), 1020 (s), 825 (m) cm^–1^. ^1^H NMR (400 MHz, *d*_6_-DMSO): *δ* = 8.23 (s, 3H, NH_3_), 3.94 (t, *J* = 3.7 Hz, 1H, CH), 3.78 (qd, *J* = 11.5, 3.8 Hz, 2H, CH_2_) ppm. ^13^C NMR (100 MHz, *d*_6_-DMSO): *δ* = 169.62, 59.64, 54.52 ppm.

**Aspartate nitrate ([Asp]NO_3_).** The similar procedure was followed as that described above for the preparation of [Gly]NO_3_. Aspartic acid (1.333 g, 10 mmol) and equivalent mole of nitric acid solution (65%) were reacted in deionized water to give [Asp]NO_3_ (1.942 g, 99%) as a colorless viscous liquid. IR (KBr, 25 °C): *ν* = 3446 (m), 3192 (s), 2937 (s), 1720 (s), 1608 (s), 1574 (s), 1381 (vs), 1068 (s), 827 (m), 631 (m) cm^–1^. ^1^H NMR (400 MHz, *d*_6_-DMSO): *δ* = 8.25 (s, 3H, NH_3_), 4.17 (s, 1H, CH), 2.88–2.76 (m, 2H, CH_2_) ppm. ^13^C NMR (100 MHz, *d*_6_-DMSO): *δ* = 170.96, 169.95, 48.61, 34.27 ppm.

**Valinium nitrate ([Val]NO_3_).** The similar procedure was followed as that described above for the preparation of [Gly]NO_3_**.** Valine (1.171 g, 10 mmol) and equivalent mole of nitric acid solution (65%) were reacted in deionized water to give [Val]NO_3_ (1.754 g, 97%) as a white solid. IR (KBr, 25 °C): *ν* = 3440 (s), 3089 (s), 1728 (s), 1479 (s), 1385 (vs), 1203 (s), 808 (m) cm^–1^. ^1^H NMR (400 MHz, *d*_6_-DMSO): *δ* = 8.21 (s, 3H, CH_3_), 3.77 (s, 1H, CH), 2.21–2.06 (m, 1H, CH), 0.97 (dd, *J* = 6.9, 5.0 Hz, 6H, CH_3_, CH_3_) ppm. ^13^C NMR (100 MHz, *d*_6_-DMSO): *δ* = 169.45, 64.31, 57.50, 18.51 ppm.

**Isoleucinium nitrate ([Ile]NO_3_).** The similar procedure was followed as that described above for the preparation of [Gly]NO_3_**.** Isoleucine (1.314 g, 10 mmol) and equivalent mole of nitric acid solution (65%) were reacted in deionized water to give [Ile]NO_3_ (1.927 g, 99%) as a white solid. IR (KBr, 25 °C): *ν* = 3440 (s), 3120 (s), 2970 (s), 1740 (s), 1614 (m), 1498 (m), 1385 (vs), 1248 (m), 1113 (m), 837 (m) cm^–1^. ^1^H NMR (400 MHz, *d*_6_-DMSO): *δ* = 8.11 (s, 3H, NH_3_), 4.17–4.08 (m, 1H, CH), 3.75 (d, *J* = 3.8 Hz, 1H, CH), 1.20 (d, *J* = 6.5 Hz, 3H, CH_3_) ppm. ^13^C NMR (100 MHz, *d*_6_-DMSO): *δ* = 169.73, 64.87, 57.50, 18.51 ppm.

**Leucinium nitrate (****[Leu]NO_3_).** The similar procedure was followed as that described above for the preparation of [Gly]NO_3_**.** Leucine (1.314 g, 10 mmol) and equivalent mole of nitric acid solution (65%) were reacted in deionized water to give [Leu]NO_3_ (1.925 g, 99%) as a white solid. IR (KBr, 25 °C): *ν* = 3434 (s), 3108 (s), 2970 (s), 2920 (s), 1724 (s), 1631 (m), 1383 (vs), 1209 (m), 1116 (m), 1033 (m), 815 (m) cm^–1^. ^1^H NMR (400 MHz, DMSO) δ 8.23 (s, 3H), 3.84 (s, 1H), 1.74 (dq, *J* = 13.3, 6.7 Hz, 1H), 1.68–1.53 (m, 2H), 0.89 (dd, *J* = 6.5, 2.9 Hz, 6H) ppm. ^13^C NMR (100 MHz, DMSO) δ 171.50 (s), 50.69 (s), 39.28 (s), 23.85 (s), 22.27 (s), 21.98 (s) ppm.

**Proline nitrate ([Pro]NO_3_).** The similar procedure was followed as that described above for the preparation of [Gly]NO_3_**.** Proline (1.158 g, 10 mmol) and equivalent mole of nitric acid solution (65%) were reacted in deionized water at 60 °C to give [Pro]NO_3_ (1.728 g, 97%) as a colorless liquid. IR (KBr, 25 °C): *ν* = 3417 (s), 2995 (vs), 1747 (vs), 1564 (m), 1390 (vs), 1213 (s), 1041 (m), 827 (m) cm^–1^. ^1^H NMR (400 MHz, *d*_6_-DMSO): *δ* = 9.40 (s, 1H, NH_2_), 8.74 (s, 1H, NH_2_), 4.30 (s, 1H, CH), 3.32–3.09 (m, 2H, CH_2_), 2.24 (m, 1H, CH_2_), 2.04–1.83 (m, 3H, CH_2_, CH_2_) ppm. ^13^C NMR (100 MHz, *d*_6_-DMSO): *δ* = 171.40, 59.87, 46.49, 28.80, 24.09 ppm.

**Figure S1.** Protonation Reactions of the Ions in [AA]ClO_4_ and [AA]NO_3_.


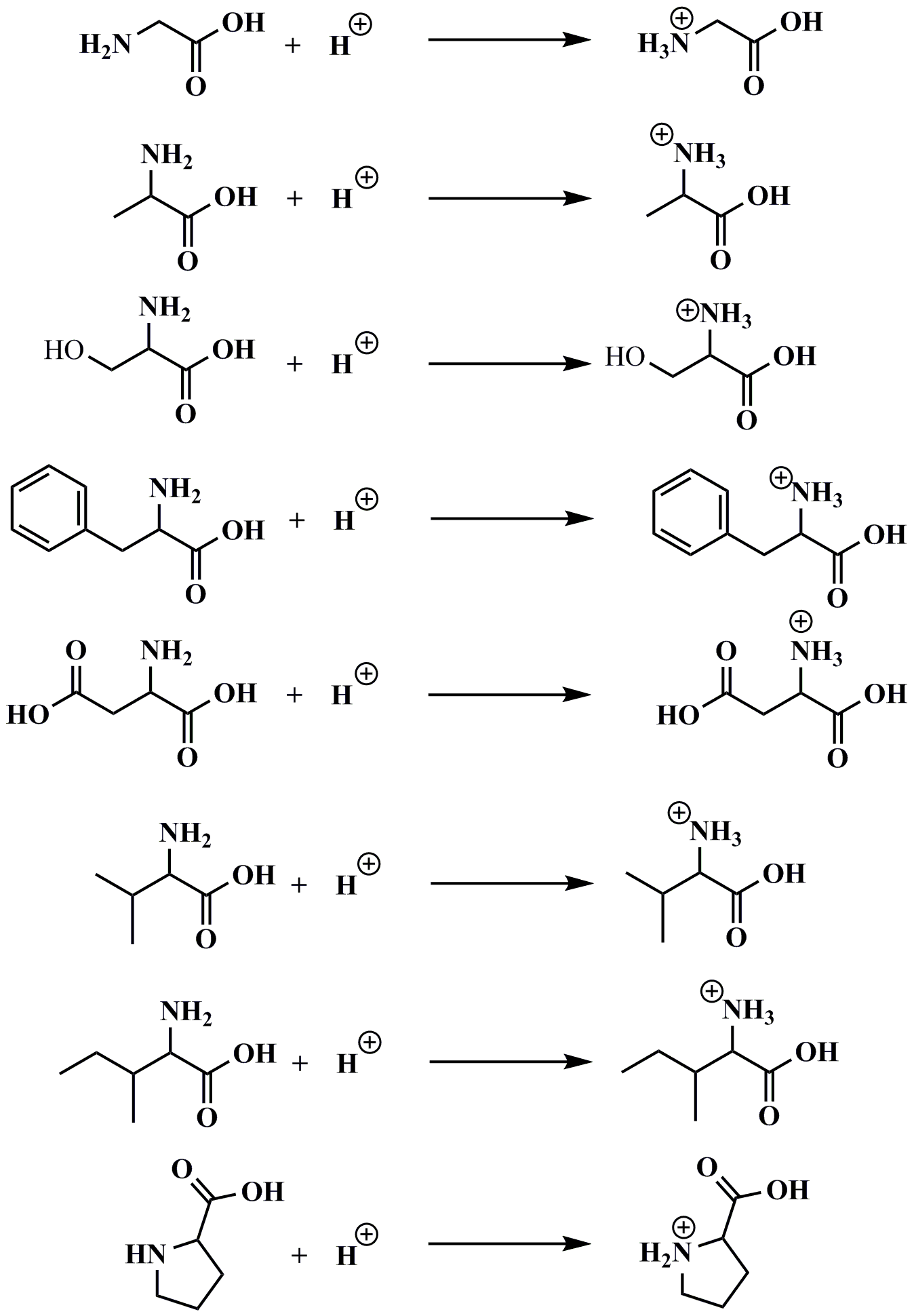


**Figure S2.** Born–Haber Cycle for Heats of Formation of [AA]ClO_4_ and [AA]NO_3_.


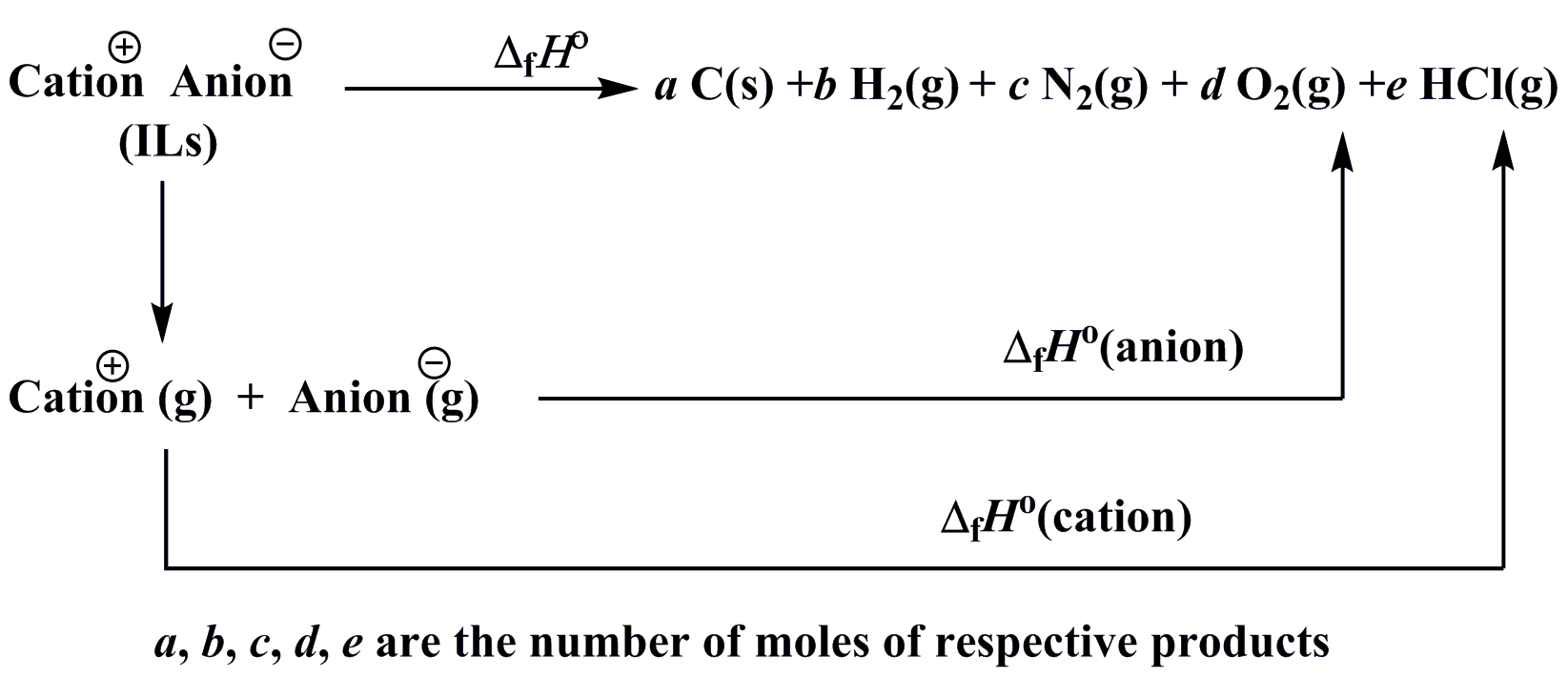


**Figure S3** Combustion Reaction Equations of [AA]ClO_4_ and [AA]NO_3_.


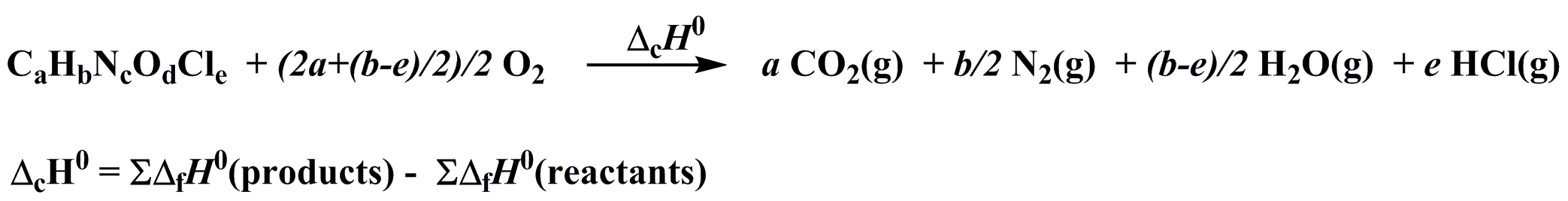


**Figure S4** TGA curves of [AA]X.


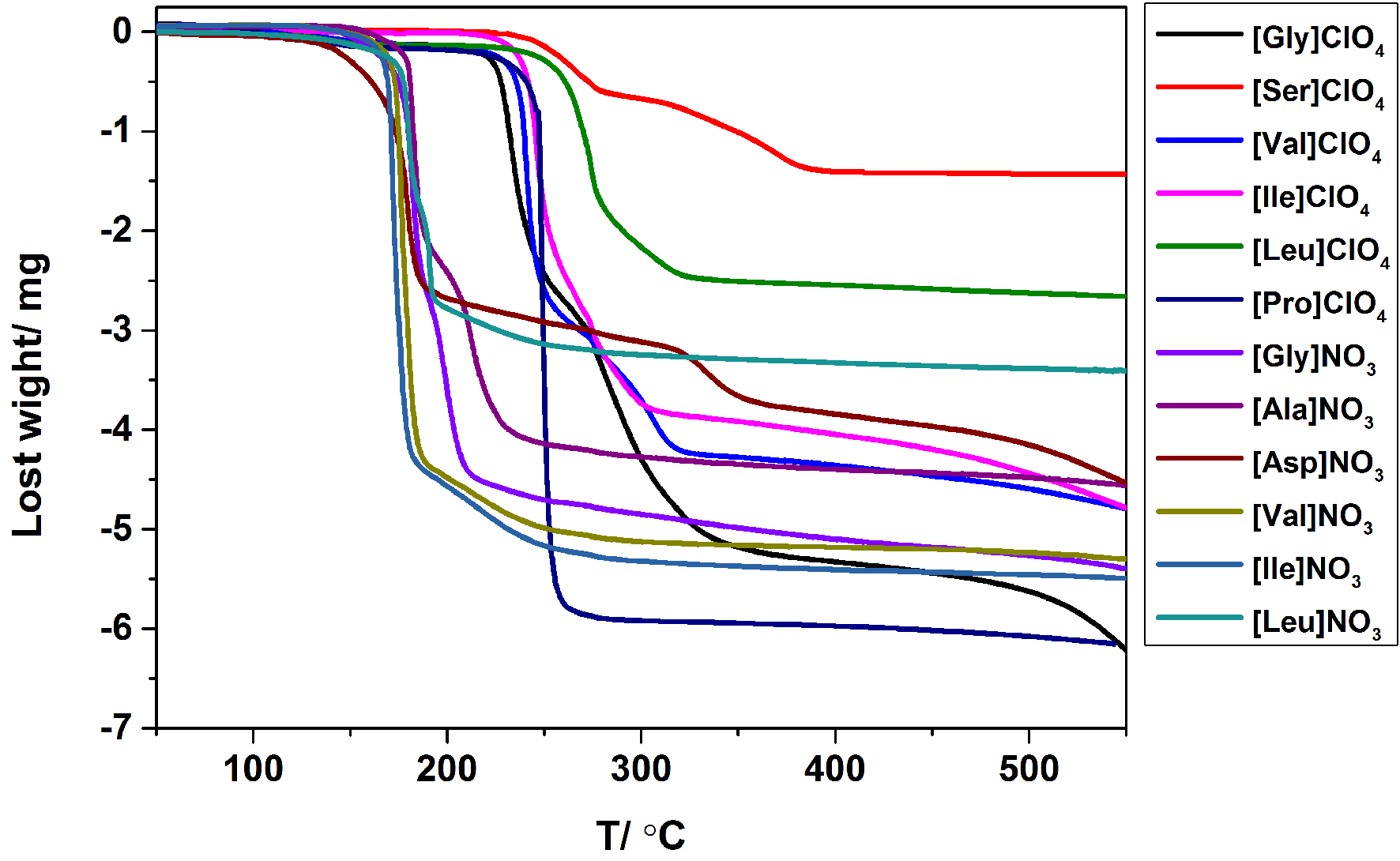


**Figure S5** DTA curves of [AA]X.


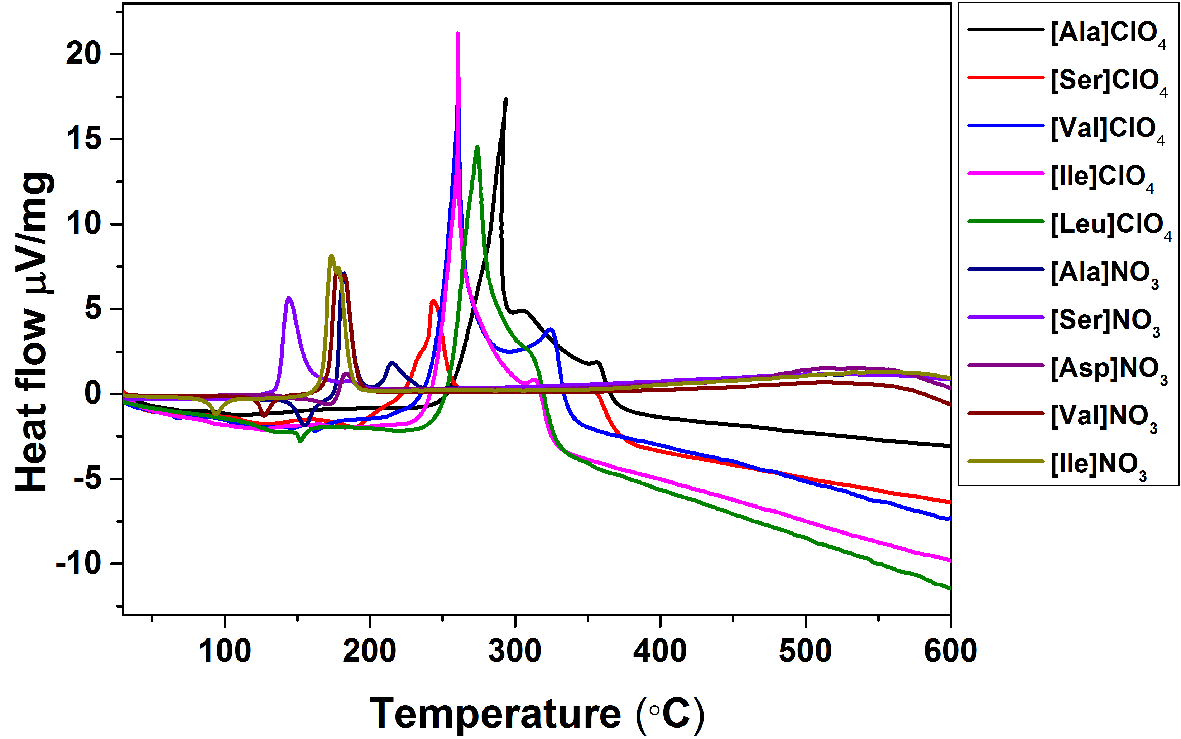


**Figure S6** Selected frames of the combustion of [Gly]ClO_4_.


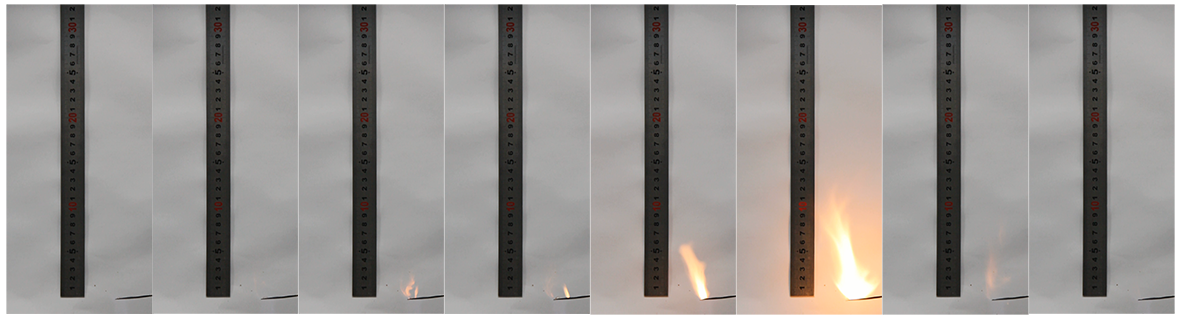


**Figure S7** Selected frames of the combustion of [Ala]ClO_4_.


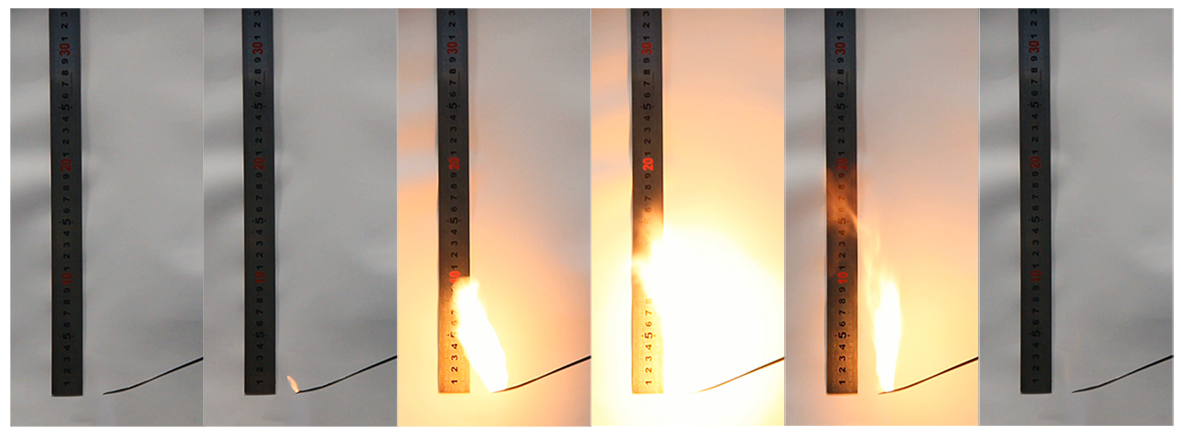


**Figure S8** Selected frames of the combustion of [Val]ClO_4_.


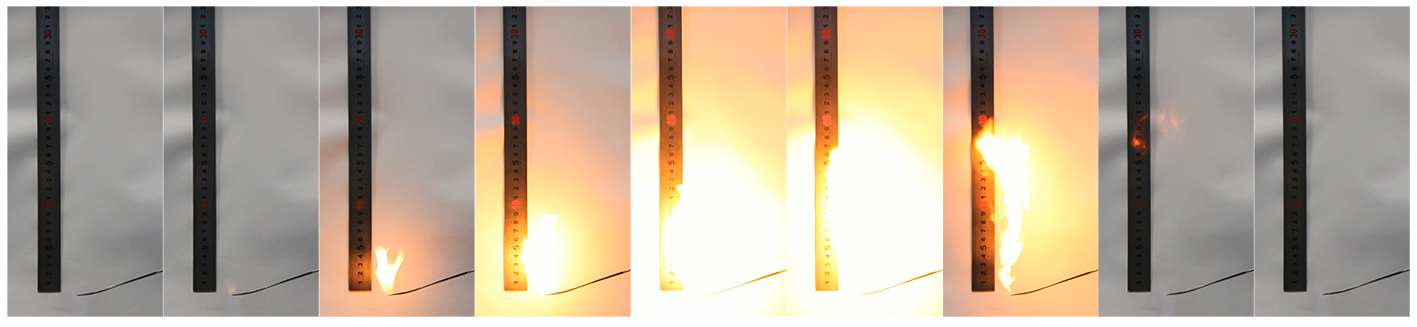


**Figure S9** Selected frames of the combustion of [Pro]ClO_4_.


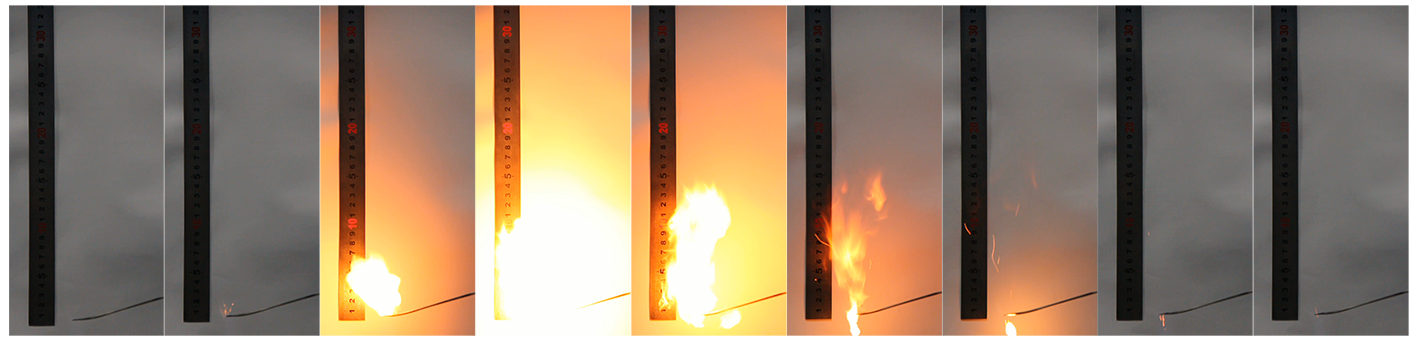


**Figure S10** Selected frames of the combustion of [Leu]ClO_4_.


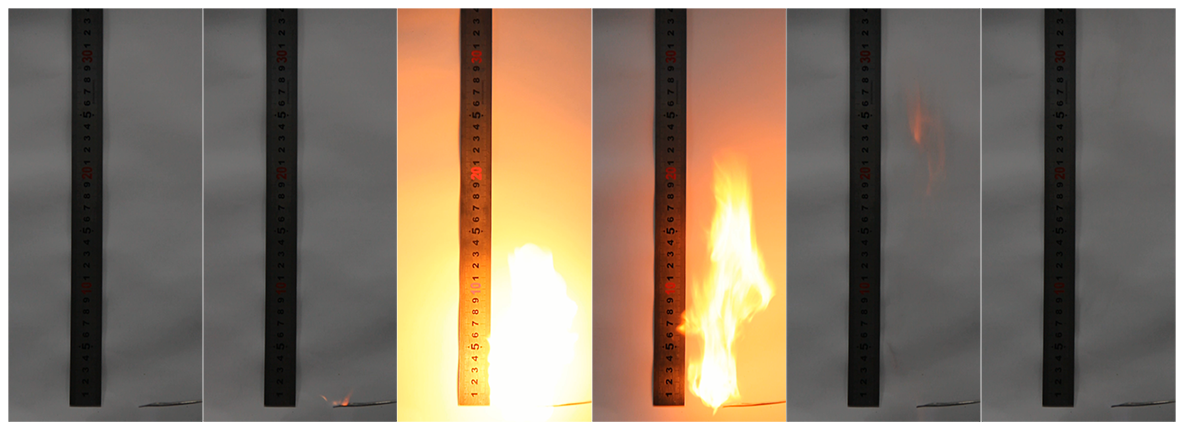


**Figure S11** Selected frames of the combustion of [Val]NO_3_.

**
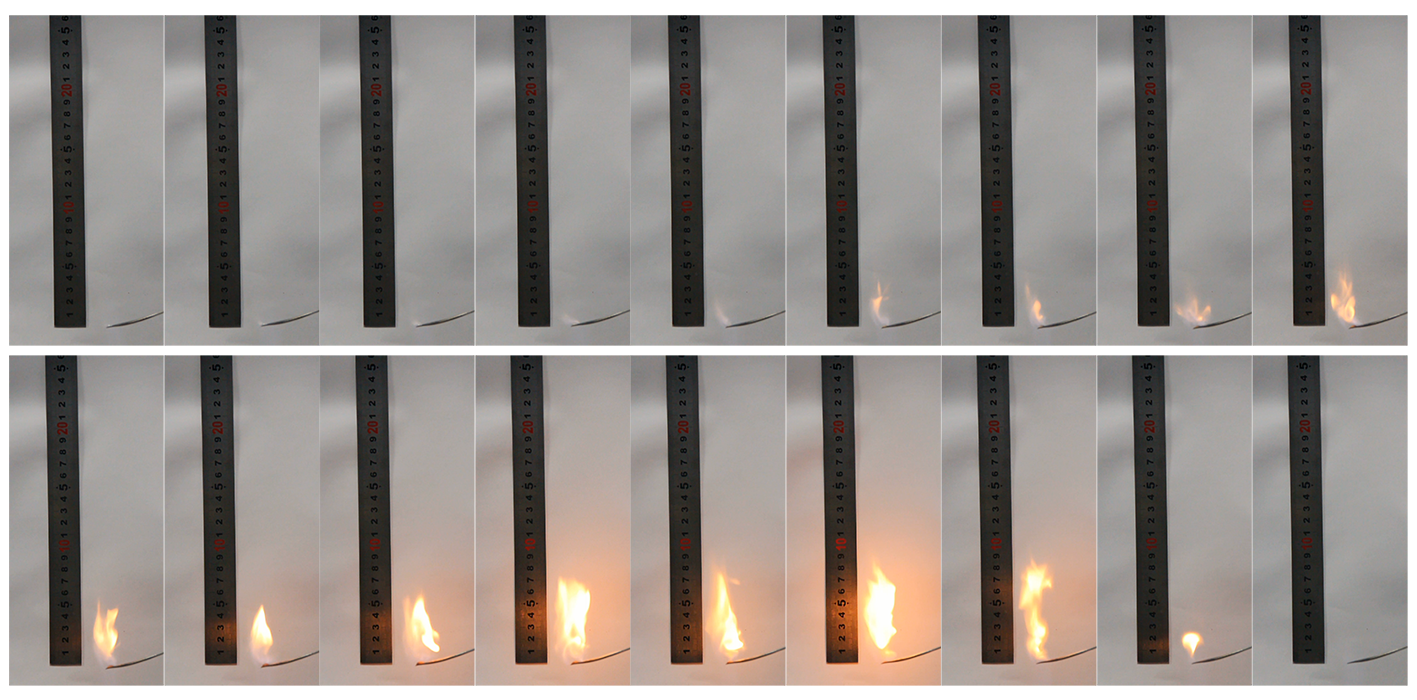
**

**Figure S12** Selected frames of the combustion of [Ile]NO_3_.


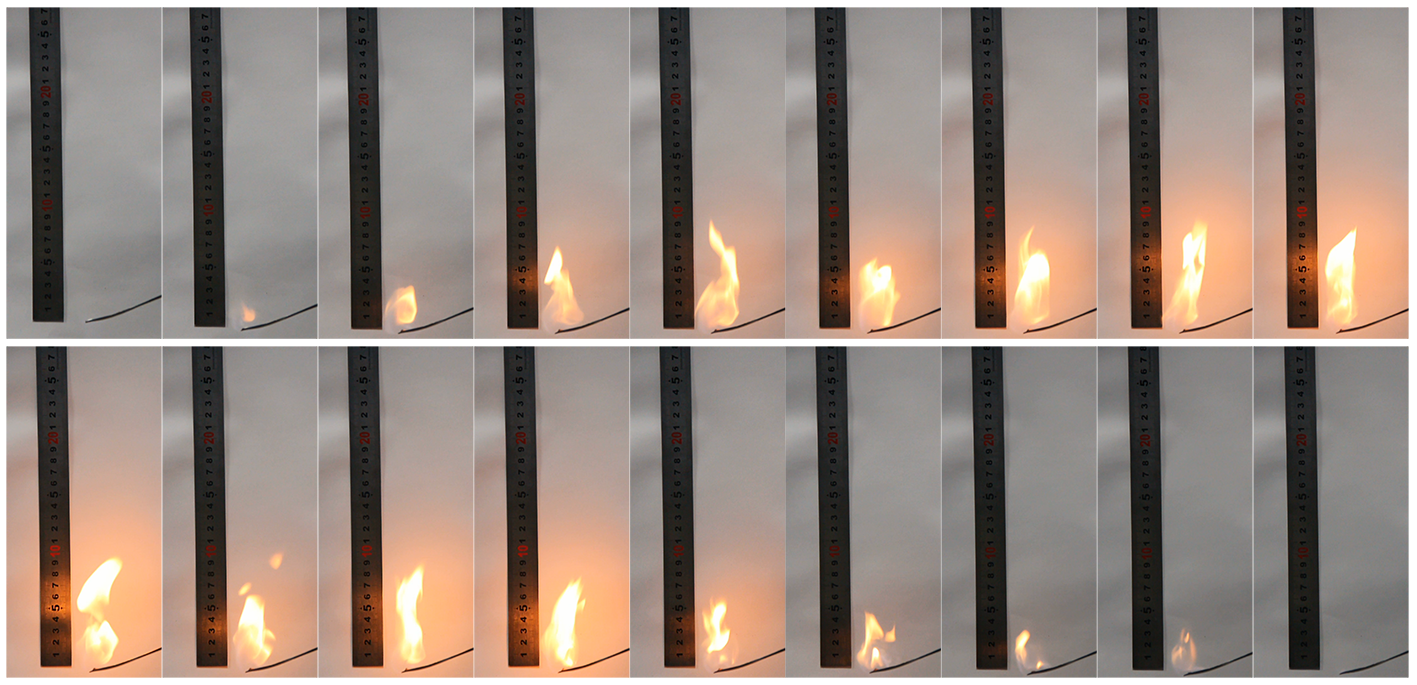


**Figure S13** Selected frames of the combustion of [Leu]NO_3_.


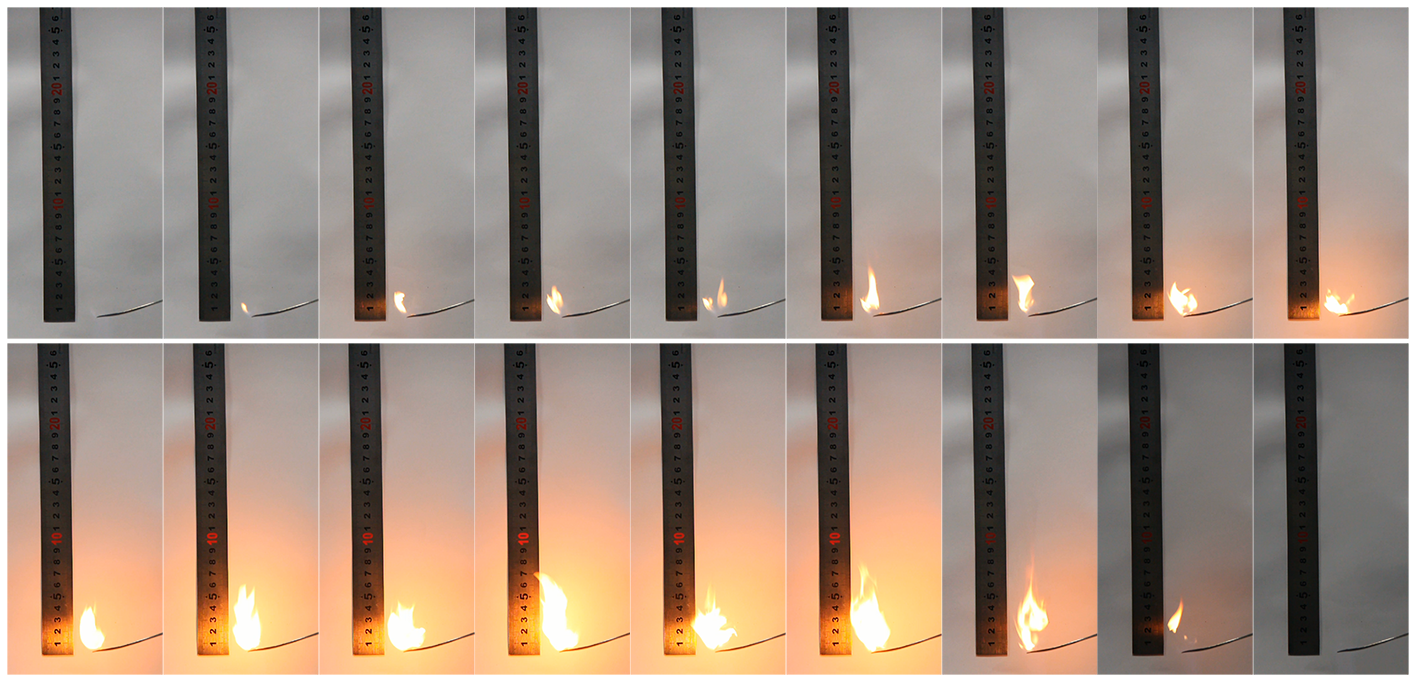


**Table S1.** Selected Bond Lengths (Å) for [Gly]ClO_4_.

| Atom | Atom | Length/Å | Atom | Atom | Length/Å |
| --- | --- | --- | --- | --- | --- |
| Cl6 | O8 | 1.4457(14) | O5 | C3 | 1.204(2) |
| Cl6 | O7 | 1.4349(15) | O4 | C3 | 1.310(2) |
| Cl6 | O10 | 1.4286(15) | N1 | C2 | 1.471(2) |
| Cl6 | O9 | 1.4223(16) | C3 | C2 | 1.502(2) |

**Table S2.** Selected Bond Angles (˚) for [Gly]ClO_4_.

| Atom | Atom | Atom | Angle/˚ | Atom | Atom | Atom | Angle/˚ |
| --- | --- | --- | --- | --- | --- | --- | --- |
| O7 | Cl6 | O8 | 108.82(9) | O9 | Cl6 | O10 | 111.72(11) |
| O10 | Cl6 | O8 | 108.92(9) | O5 | C3 | O4 | 124.96(17) |
| O10 | Cl6 | O7 | 109.29(11) | O5 | C3 | C2 | 123.35(16) |
| O9 | Cl6 | O8 | 109.16(10) | O4 | C3 | C2 | 111.69(15) |
| O9 | Cl6 | O7 | 108.89(10) | N1 | C2 | C3 | 110.39(15) |

**Table S4.** Selected Bond Lengths (Å) for [Ala]NO_3_.

| Atom | Atom | Length/Å | Atom | Atom | Length/Å |
| --- | --- | --- | --- | --- | --- |
| O1 | C4 | 1.2123(7) | C4 | O6 | 1.300(6) |
| N2 | O3 | 1.2146(7) | C4 | C9 | 1.491(7) |
| N2 | O5 | 1.252(6) | N8 | C9 | 1.482(6) |
| N2 | O7 | 1.215(7) | C9 | C10 | 1.524(8) |

**Table S5.** Selected Bond Angles (˚) for [Ala]NO_3_ .

| Atom | Atom | Atom | Angle/˚ | Atom | Atom | Atom | Angle/˚ |
| --- | --- | --- | --- | --- | --- | --- | --- |
| O3 | N2 | O5 | 117.2(5) | O6 | C4 | C9 | 111.9(4) |
| O3 | N2 | O5 | 117.2(5) | O6 | C4 | C9 | 111.2(4) |
| O3 | N2 | O5 | 121.4(5) | O6 | C4 | C9 | 109.7(4) |
| O1 | C4 | O6 | 124.5(5) | O6 | C4 | C9 | 109.1(5) |
| O1 | C4 | C9 | 123.6(5) |  |  |  |  |

**The geometry coordinates (Å) of the four cluster conformers in [Gly]ClO_4_.**

**N1–H1A–O7**

Cl 4.76950000 6.17110000 0.76620000

O 4.68600000 5.00400000 1.61440000

O 3.44790000 6.68390000 0.55300000

O 5.33600000 5.79160000 -0.48830000

O 5.54970000 7.16600000 1.41550000

O 4.46410000 6.84610000 -3.50680000

O 3.95450000 4.79740000 -4.22440000

H 4.64230000 4.88440000 -4.66280000

N 2.19370000 7.07190000 -2.05750000

H 2.83010000 7.19920000 -1.44790000

H 2.19850000 7.74620000 -2.63690000

H 1.40150000 7.03360000 -1.65450000

C 3.73390000 5.88920000 -3.53700000

C 2.44390000 5.81080000 -2.77000000

H 2.48810000 5.08080000 -2.13320000

H 1.71290000 5.63270000 -3.38260000

**N1–H1B–O5**

O 0.71180000 9.38650000 3.50680000

O 1.22140000 11.43520000 4.22440000

H 0.53360000 11.34820000 4.66280000

N 2.98220000 9.16070000 2.05750000

H 2.34580000 9.03340000 1.44790000

H 2.97740000 8.48640000 2.63690000

H 3.77440000 9.19900000 1.65450000

C 1.44200000 10.34340000 3.53700000

C 2.73200000 10.42180000 2.77000000

H 2.68780000 11.15180000 2.13320000

H 3.46300000 10.59990000 3.38260000

O 2.91880000 6.84610000 3.84660000

O 2.40920000 4.79740000 3.12890000

H 3.09700000 4.88440000 2.69060000

N 0.64840000 7.07190000 5.29590000

H 1.28480000 7.19920000 5.90550000

H 0.65320000 7.74620000 4.71650000

H -0.14380000 7.03360000 5.69890000

C 2.18860000 5.88920000 3.81640000

C 0.89860000 5.81080000 4.58340000

H 0.94280000 5.08080000 5.22020000

H 0.16760000 5.63270000 3.97080000

**N1–H1C–O10**

Cl 4.76950000 6.17110000 0.76620000

O 4.68600000 5.00400000 1.61440000

O 3.44790000 6.68390000 0.55300000

O 5.33600000 5.79160000 -0.48830000

O 5.54970000 7.16600000 1.41550000

O 9.64000000 6.84610000 -3.50680000

O 9.13040000 4.79740000 -4.22440000

H 9.81820000 4.88440000 -4.66280000

N 7.36960000 7.07190000 -2.05750000

H 8.00600000 7.19920000 -1.44790000

H 7.37440000 7.74620000 -2.63690000

H 6.57740000 7.03360000 -1.65450000

C 8.90980000 5.88920000 -3.53700000

C 7.61980000 5.81080000 -2.77000000

H 7.66400000 5.08080000 -2.13320000

H 6.88880000 5.63270000 -3.38260000

**O4–H4–O8**

Cl 4.76950000 6.17110000 0.76620000

O 4.68600000 5.00400000 1.61440000

O 3.44790000 6.68390000 0.55300000

O 5.33600000 5.79160000 -0.48830000

O 5.54970000 7.16600000 1.41550000

O 2.91880000 6.84610000 3.84660000

O 2.40920000 4.79740000 3.12890000

H 3.09700000 4.88440000 2.69060000

N 0.64840000 7.07190000 5.29590000

H 1.28480000 7.19920000 5.90550000

H 0.65320000 7.74620000 4.71650000

H -0.14380000 7.03360000 5.69890000

C 2.18860000 5.88920000 3.81640000

C 0.89860000 5.81080000 4.58340000

H 0.94280000 5.08080000 5.22020000

H 0.16760000 5.63270000 3.97080000

**The geometry coordinates (Å) of the four cluster conformers in [Ala]NO_3_.**

**N8–H8C–O3**

O -0.4709 4.6842 8.3667

C 0.5965 4.1063 8.3554

O 1.2038 3.6598 9.4138

N 0.952 4.7088 6.0274

H 1.2157 5.5308 6.242

H 0.0674 4.6984 5.9258

H 1.3402 4.4617 5.2659

C 1.3374 3.7681 7.1067

H 2.299 3.8539 7.2745

C 1.0386 2.3436 6.6566

H 0.107 2.2652 6.4404

H 1.2565 1.7321 7.3632

H 1.5655 2.136 5.8806

H 0.7358 3.6658 10.2124

N 3.7213 3.8651 4.2253

O 2.5542 3.8047 3.7946

O 3.8826 4.3236 5.3805

O 4.6647 3.4956 3.5542

**O6–H6–O3**

O 2.359 -0.9512 7.7666

C 3.4264 -0.3733 7.7779

O 4.0337 0.0732 6.7195

N 3.7818 -0.9758 10.1059

H 4.0456 -1.7978 9.8913

H 2.8972 -0.9654 10.2075

H 4.1701 -0.7287 10.8674

C 4.1672 -0.0351 9.0266

H 5.1288 -0.1209 8.8588

C 3.8684 1.3894 9.4767

H 2.9368 1.4678 9.6929

H 4.0863 2.0009 8.7701

H 4.3953 1.597 10.2527

H 3.5656 0.0672 5.9209

N 1.9384 0.1321 3.8413

O 3.1055 0.0717 4.2721

O 1.7771 0.5906 2.6862

O 0.995 -0.2374 4.5125

**N8–H8B–O5**

O 3.3007 2.7818 16.4334

C 2.2333 3.3597 16.4221

O 1.626 3.8062 17.4804

N 1.8779 2.7572 14.0941

H 1.6141 1.9352 14.3086

H 2.7625 2.7676 13.9924

H 1.4896 3.0043 13.3326

C 1.4925 3.6979 15.1734

H 0.5309 3.6121 15.3412

C 1.7913 5.1224 14.7232

H 2.7229 5.2008 14.5071

H 1.5734 5.7339 15.4299

H 1.2644 5.33 13.9472

H 2.0941 3.8002 18.279

N 4.7683 3.6009 12.292

O 5.9353 3.6613 11.8612

O 4.607 3.1424 13.4471

O 3.8248 3.9704 11.6208

**N8–H8A–O1**

O -0.4709 -2.7826 -7.7666

C 0.5965 -3.3597 -7.7779

O 1.2033 -3.8062 -6.7195

N 0.952 -2.7572 -10.1059

H 1.2163 -1.9344 -9.8913

H 0.0674 -2.7676 -10.2075

H 1.3402 -3.0043 -10.8674

C 1.3374 -3.6972 -9.0266

H 2.299 -3.6121 -8.8588

C 1.0386 -5.1224 -9.4767

H 0.107 -5.2008 -9.6945

H 1.2553 -5.7339 -8.7701

H 1.566 -5.3307 -10.2527

H 0.7358 -3.8002 -5.9048

O -3.3007 -0.9504 -8.3667

C -2.2333 -0.3733 -8.3554

O -1.6266 0.0732 -9.4138

N -1.8779 -0.9758 -6.0274

H -1.6136 -1.7986 -6.242

H -2.7625 -0.9654 -5.9258

H -1.4896 -0.7287 -5.2659

C -1.4925 -0.0358 -7.1067

H -0.5309 -0.1209 -7.2745

C -1.7913 1.3894 -6.6566

H -2.7229 1.4678 -6.4388

H -1.5745 2.0009 -7.3632

H -1.2638 1.5977 -5.8806

H -2.0941 0.0672 -10.2285

**The optimized geometry coordinates of 8 amino acid cations**

Gly^+^

C -0.59987300 -0.81742600 0.00000000

C 0.60975200 0.11024700 -0.00004200

H -0.62131600 -1.44610500 0.89167000

H -0.62134900 -1.44612000 -0.89165900

O 0.47866400 1.31280900 -0.00002000

O 1.73111800 -0.59176500 0.00002600

H 2.48796300 0.01866600 0.00005200

H -2.68193000 -0.47662800 0.00001200

H -1.80617000 0.68508900 0.81720300

H -1.80617400 0.68510400 -0.81716500

N -1.81265100 0.06495900 0.00001300

Ala^+^

C 0.60818400 0.18089500 0.40864400

H 0.66775900 0.30128000 1.49468200

C 1.17508900 1.39279400 -0.31619300

C -0.83522700 -0.15957400 0.03656500

H 1.08208900 1.27900100 -1.40087300

H 0.61125900 2.27769400 -0.01781800

H 2.22498100 1.54955100 -0.05479400

O -1.15739400 -1.27450300 -0.31207600

O -1.61918300 0.90005000 0.16763300

H -2.53165400 0.64212900 -0.04780300

H 2.27881900 -1.11215600 0.58370000

H 0.81998200 -1.89390900 0.26928800

H 1.60651100 -1.09356600 -0.93342300

N 1.39494200 -1.06272700 0.06978400

Ser^+^

N -0.34148500 1.89484800 -0.25821900

H -0.84510200 1.85309300 0.64488600

C 0.18741900 0.51127200 -0.52292100

H 0.34954600 0.37980300 -1.59353300

C -0.89627100 -0.42071700 0.02495900

O -1.58025100 -0.06133800 0.96198200

C 1.49116600 0.33796500 0.26721000

H 2.26018400 0.99141600 -0.16846900

H 1.31355200 0.63476900 1.31250600

O 1.80772500 -1.02279300 0.14830600

H 2.67282000 -1.18065700 0.54167000

O -0.95515400 -1.56192500 -0.62989700

H -1.61645200 -2.13323500 -0.20245900

H -1.01738000 2.18849800 -0.97067700

H 0.40077400 2.59969800 -0.21500400

Asp^+^

N -1.79885700 -1.56083900 -0.01426400

H -1.69427900 -2.45231700 0.47806100

C -0.66651600 -0.60806200 0.28263300

H -0.41859700 -0.72065300 1.34197800

C -1.26483200 0.78301400 0.05707500

O -2.45288300 0.94083700 -0.12207700

C 0.54690800 -0.92245000 -0.59023500

H 0.78301500 -1.99146700 -0.51414400

H 0.35978400 -0.67513100 -1.63927400

C 1.79543200 -0.16566600 -0.14805800

O 1.91926600 -0.23814000 1.19603600

O 2.56297800 0.37180600 -0.89861900

O -0.32420200 1.71267600 0.09760600

H -0.73256500 2.58553200 -0.03561700

H 2.73855800 0.22080300 1.44276600

H -1.86559100 -1.75446300 -1.01947700

H -2.68555000 -1.10488100 0.25349700

Val^+^

N 0.68426400 -1.73632900 -0.68304100

H 1.19355900 -1.82333700 0.21273000

C 0.12926300 -0.33155500 -0.74877200

H 0.12127100 -0.02005000 -1.79641400

C 1.13251100 0.49432900 0.05571000

O 1.92202700 -0.03265900 0.81215000

C -1.29909000 -0.31177300 -0.16869700

H -1.85161500 -1.07672300 -0.73563100

C -1.32276100 -0.67368700 1.31933100

H -2.35883900 -0.72459400 1.66253800

H -0.86543500 -1.64535000 1.54158700

H -0.81071000 0.08625700 1.91839700

C -1.98077400 1.03397300 -0.43256700

H -3.03436100 0.95750700 -0.15290900

H -1.52928900 1.83284000 0.15922700

H -1.92874400 1.31217200 -1.48929700

O 0.99891700 1.79213500 -0.17367500

H 1.64761700 2.26927600 0.37239500

H -0.05765500 -2.43967700 -0.73944000

H 1.36190400 -1.91754700 -1.42972500

Ile^+^

N 1.41331200 -1.59763800 -0.69906900

H 1.97348500 -1.56608800 0.17020000

C 0.58910700 -0.32987200 -0.74424400

H 0.49286800 -0.02667000 -1.79002700

C -0.80045700 -0.58986800 -0.12865000

C 1.43037400 0.67778000 0.03873800

H -1.19887100 -1.46429600 -0.66921900

C -0.70639900 -0.91905700 1.36447100

C -1.75188100 0.58181000 -0.41980300

O 2.33166300 0.31681200 0.76766800

H -0.41108800 -0.03592600 1.94100600

H -1.67649800 -1.25329700 1.73599800

H 0.00795800 -1.72070400 1.58870900

H -1.42232400 1.46770400 0.13189700

H -1.68873900 0.83007100 -1.48667700

C -3.20382100 0.25548100 -0.06258500

H -3.33916500 0.12869200 1.01396200

H -3.85810200 1.06985900 -0.38202000

H -3.53636200 -0.65879000 -0.56466000

O 1.03503000 1.92399300 -0.17409700

H 1.59246700 2.51771400 0.35831700

H 2.07401400 -1.65543400 -1.47976000

H 0.82208600 -2.43344300 -0.71036800

Leu^+^

N -1.72502200 -1.64068100 0.49289200

H -2.47043300 -1.55949000 1.19190300

C -0.75278400 -0.48439300 0.54972200

H -0.57796000 -0.24096700 1.59908700

C 0.53444600 -0.90405900 -0.16899800

C -1.47533600 0.64426900 -0.18117500

H 0.94624000 -1.77612800 0.36066600

H 0.27178500 -1.22188400 -1.18828400

C 1.62353800 0.17825500 -0.23019200

O -2.32502300 0.40570000 -1.01371600

H 1.23773400 1.02889400 -0.80490600

C 2.01908500 0.65861500 1.16786600

C 2.83306100 -0.39324200 -0.97528000

H 2.33775000 -0.18717300 1.78955900

H 2.86092300 1.35292100 1.10044500

H 1.20789200 1.18682700 1.67844700

H 2.56051100 -0.74144700 -1.97650500

H 3.60754800 0.37066600 -1.08155900

H 3.26454500 -1.23467200 -0.42141500

O -1.04130800 1.83815000 0.19693300

H -1.50623800 2.51442700 -0.32582200

H -1.24903700 -2.53761000 0.62561300

H -2.17752000 -1.61706800 -0.43487800

Pro^+^

C 1.92563700 -0.74015900 -0.34157100

C -0.12148300 0.22310800 0.60438300

H 1.64203400 -0.99323300 -1.36551900

H 2.75865500 -1.36628400 -0.02095600

C 2.10711500 0.76099100 -0.16197400

C 0.66372700 1.27625400 -0.20356400

H -0.23382200 0.51012200 1.65239900

C -1.47234000 -0.13017100 -0.00234000

H 2.73075100 1.18723200 -0.94994200

H 2.57462900 0.97914400 0.80422400

H 0.30109300 1.30301900 -1.23623200

H 0.53701700 2.26923500 0.22961900

O -1.68819500 -1.21327900 -0.50369900

H 0.11605300 -1.77450800 0.12739700

H 1.01032000 -1.33392600 1.46372900

N 0.71923100 -1.03611800 0.52985700

O -2.30411300 0.89841300 0.07325600

H -3.14880700 0.65081500 -0.33978300

**The optimized geometry coordinates of 8 amino acids.**

Gly

N 1.96876700 0.00892300 -0.00005000

C 0.72269600 -0.73069500 0.00010000

H 0.67923500 -1.38662700 -0.87503100

H 0.67928900 -1.38629300 0.87549400

C -0.54106700 0.11473600 0.00001100

O -0.58210400 1.32407100 0.00003300

O -1.64804200 -0.66929300 -0.00006100

H -2.40636500 -0.06723300 -0.00010500

H 2.00897400 0.61737500 -0.81128100

H 2.00888200 0.61785100 0.81083000

Ala

N -1.55786400 -0.98971100 -0.35056400

H -1.55739800 -1.36749300 0.59350500

C -0.65791000 0.15031300 -0.40973200

H -0.63230100 0.52427600 -1.43844400

C 0.77381400 -0.17926300 -0.00783400

O 1.11599500 -1.13768900 0.64903600

C -1.17371200 1.26163100 0.50988400

H -0.54417900 2.15173000 0.44313900

H -2.19620200 1.51544300 0.22286000

H -1.18038800 0.91375700 1.54865600

O 1.64254200 0.76548100 -0.44980200

H -1.21951100 -1.73655100 -0.95081900

H 2.51357900 0.50838900 -0.11273100

Ser

N 0.25108300 2.00831600 -0.22697600

H -0.00757300 2.17060400 0.74330400

C 0.28270700 0.57148800 -0.48077900

H 0.33832600 0.39438800 -1.55728400

C -0.92740400 -0.15672500 0.08775500

O -1.31614000 -0.01588700 1.22791900

C 1.52631400 -0.00143900 0.18844400

H 2.40952700 0.45689100 -0.26926900

H 1.49536100 0.25715100 1.25534100

O 1.49576200 -1.41177400 -0.00061700

H 2.22736100 -1.78973500 0.49410100

O -1.52949800 -0.95811300 -0.81509600

H -2.25525800 -1.39579200 -0.34463900

H -0.45601100 2.45452700 -0.80288900

Asp

N -1.93886100 1.54485500 -0.18850700

H -2.37623100 1.31595700 0.70032400

C -0.76349800 0.71282800 -0.38441400

H -0.41951500 0.80193000 -1.41800000

C -1.16480800 -0.72662700 -0.12485300

O -1.96757000 -1.07126700 0.71195700

C 0.42120100 1.04710700 0.56260600

H 0.69871200 2.09263000 0.39432000

H 0.10699000 0.91269500 1.60065700

C 1.62248000 0.17200900 0.30567100

O 2.19828200 0.48239000 -0.88345900

O 2.02352200 -0.71124100 1.02965000

O -0.46274600 -1.58356700 -0.89897700

H -0.70071500 -2.47561100 -0.60273200

H 2.92089500 -0.15102000 -1.00611600

H -1.68226100 2.52701100 -0.17632200

Val

N -0.39538300 1.81910200 -0.90122300

H -0.59773100 2.20813300 0.01552900

C -0.10967800 0.39303900 -0.77777300

H -0.20216600 -0.07063200 -1.76595300

C -1.18158900 -0.20131000 0.11190500

O -1.66801100 0.35877300 1.07031700

C 1.30344300 0.04495100 -0.23538100

H 1.99144300 0.55628600 -0.92530300

C 1.55283600 0.58300700 1.17414200

H 2.59346100 0.40253700 1.46205300

H 1.36869000 1.65920600 1.24024900

H 0.90871000 0.08456500 1.90602000

C 1.59600300 -1.45559000 -0.30972200

H 2.65237000 -1.64424200 -0.09329000

H 0.99926400 -2.01098900 0.42032300

H 1.37123500 -1.85784800 -1.30282100

O -1.52133200 -1.45804000 -0.26176700

H -2.18383300 -1.75479700 0.38090500

H 0.41488400 2.30362100 -1.27658400

Ile

N 1.35273400 -1.64517100 -0.90544400

H 1.65410800 -1.96480200 0.01176900

C 0.65662900 -0.36852400 -0.76698400

H 0.58540400 0.09394400 -1.75673800

C 1.39578300 0.59933100 0.14624200

O 2.01309600 0.27808500 1.13970900

C -0.78619900 -0.59690500 -0.25815700

H -1.24085800 -1.24321200 -1.02174400

C -1.58095700 0.71487300 -0.20428500

H -1.22334900 1.32792000 0.63300300

H -1.38050400 1.29084800 -1.11632300

C -0.81373500 -1.34096400 1.07892200

H -1.84380700 -1.55397400 1.37656500

H -0.29316200 -2.30011600 1.01045800

H -0.34592600 -0.74761700 1.87202400

C -3.09041800 0.49997600 -0.07101400

H -3.62200000 1.45562800 -0.10757200

H -3.46416800 -0.12530000 -0.88886600

H -3.34939800 0.01356700 0.87316400

O 1.28706700 1.88670200 -0.26860500

H 2.20049000 -1.51382800 -1.45081500

H 1.76610400 2.41811800 0.38600800

Leu

N -1.79263500 1.66425400 -0.63669300

H -2.05372600 1.92075900 0.31302400

C -0.82145700 0.56982500 -0.57904400

H -0.65075500 0.20937200 -1.59623000

C 0.51671500 0.94861400 0.08380200

C -1.45670500 -0.54112300 0.23030400

H 0.95896100 1.76385200 -0.50823700

H 0.28424500 1.35688900 1.07694100

C 1.54787600 -0.17810200 0.21965300

O -2.00123900 -0.37338500 1.29952200

H 1.10897700 -0.98097900 0.82865500

C 1.93826400 -0.76009900 -1.14145100

C 2.78510300 0.34525900 0.95374200

H 2.31350600 0.03411300 -1.79913000

H 2.73437600 -1.50330700 -1.02665300

H 1.09525800 -1.25043600 -1.63562100

H 2.52073300 0.75281800 1.93507500

H 3.52041300 -0.45255900 1.10098500

H 3.26379600 1.14200000 0.37164500

O -1.31428500 -1.75617100 -0.34984000

H -1.72575000 -2.39116400 0.25673100

H -1.35617500 2.47906700 -1.05983100

Pro

N -0.83293900 -0.99626000 -0.73515900

C 0.09666300 0.14103900 -0.72196000

H 0.38191700 0.43818900 -1.73615800

C 1.36548200 -0.20712800 0.02670600

O 1.50340300 -1.14841500 0.77873400

C -0.63826800 1.31307400 -0.00329300

H 0.04375100 1.93775700 0.57905200

H -1.12070400 1.94562100 -0.75545200

C -1.69897800 0.59025700 0.83203500

H -1.25228900 0.17757100 1.74368400

H -2.53442900 1.23989400 1.10913400

C -2.09338500 -0.54636100 -0.11467700

H -2.76647600 -0.16687100 -0.89317200

H -2.58650500 -1.38771900 0.37926000

O 2.34626000 0.69284700 -0.22205200

H 3.10594800 0.41865800 0.31287600

H -0.42703800 -1.73002200 -0.15943100
